# Metabolic engineering of *Paracoccus denitrificans* for dual degradation of sulfamethoxazole and ammonia nitrogen

**DOI:** 10.1128/spectrum.00146-23

**Published:** 2023-09-21

**Authors:** Shenghu Zhou, Rongrong Zhu, Xiaoqian Niu, Yunying Zhao, Yu Deng

**Affiliations:** 1 National Engineering Research Center of Cereal Fermentation and Food Biomanufacturing, Jiangnan University, Wuxi, Jiangsu, China; 2 Jiangsu Provincial Research Center for Bioactive Product Processing Technology, Jiangnan University, Wuxi, Jiangsu, China; Chinese Academy of Sciences, Xicheng, Beijing, China

**Keywords:** FMN reductase, metabolic engineering, nitrogen removal, promoter, *Paracoccus denitrificans *DYTN-1, sulfamethoxazole degradation

## Abstract

Sulfamethoxazole (SMX), as one of the most widely used sulfonamide antibiotics, has been frequently detected in the aqueous environment, posing potential risks to the environment and human health. Although microbial degradation methods have been widely applied, some issues remain, including low degradation efficiency and poor environmental adaptability. In this regard, constructing efficient degrading bacteria by metabolic engineering is an ideal solution to these challenges. In this study, we used *Paracoccus denitrificans* DYTN-1, a superior nitrogen removal environment strain, as chassis to construct an SMX degradation pathway, obtaining a new bacteria for simultaneous degradation of SMX and removal of ammonia nitrogen. In doing this, we first identified and characterized four native promoters of *P. denitrificans* DYTN-1 with gradient strength to control the expression of the SMX degradation pathway. After degradation pathway expression level optimization and FMN reductase optimization, SMX degradation efficiency was significantly improved. The constructed *P. d-*pIAB_4_-P*_CS_-sutR* strain exhibited superior co-degradation of SMX and ammonia nitrogen contaminants with degradation rates of 44% and 71%, respectively. This study could pave the way for SMX degradation engineered strain design and evolution of environmental bioremediation.

**IMPORTANCE:**

The abuse of sulfamethoxazole (SMX) had led to an increased accumulation in the environment, resulting in the disruption of the structure of microbial communities, further disrupting the bio-degradation process of other pollutants, such as ammonia nitrogen. To solve this challenge, we first identified and characterized four native promoters of *Paracoccus denitrificans* DYTN-1 with gradient strength to control the expression of the SMX degradation pathway. Then SMX degradation efficiency was significantly improved with degradation pathway expression level optimization and FMN reductase optimization. Finally, the superior nitrogen removal environment strain, *P. denitrificans* DYTN-1, obtained an SMX degradation function. This pioneering study of metabolic engineering to enhance the SMX degradation in microorganisms could pave the way for designing the engineered strains of SMX and nitrogen co-degradation and the environmental bioremediation.

## INTRODUCTION

Sulfonamides are a class of broad-spectrum antibacterial drugs that prevent and treat infectious diseases in animals and humans. However, the abuse of sulfonamides had led to an increased accumulation in the environment, resulting in disruption of the structure of microbial communities, and endangering the health of humans and animals (1 – 3). Furthermore, degradation of sulfonamides in the environment is difficult and has caused resistance gene drift (4). Hence, sulfonamide pollution has posed a severe threat to the ecological environment and human health (5 – 8). In this regard, efficient removal and toxicity reduction of sulfonamides in the environment is an inevitable requirement. Sulfamethoxazole (SMX), used to treat infectious diseases, is one of the most frequently used sulfonamide antibiotics. SMX is excreted into the environment through urine or feces and is mainly present in sewage (9, 10), hospitals (11), aquaculture farms, the pharmaceutical factory waste (12), and surface water (13). Studies have shown that SMX may exhibit direct neurotoxicity. SMX has also been directly linked to side effects such as altered intestinal transmission and neuropsychiatric complications in humans (14). In addition, SMX poses a potential ecological risk to aquatic organisms due to its acute toxicity (15). Therefore, the efficient removal of SMX is becoming a keen focus of attention.

Various physical, chemical, and biological methods have been established to remove SMX from the environment. Generally, solid-phase adsorption and oxidation methods have been primarily used in physical and chemical SMX removal processes, respectively. Phadunghus et al. found that the removal rate of SMX by nanofiltration and reverse osmosis membrane separation technology could reach 94–98% (16). Yazdanbakhsh et al. used the Mn-WO_3_/LED photocatalytic system to degrade SMX in wastewater, with a removal rate of up to 85% in 3 h (17). However, both the physical and chemical methods for degradation of SMX are costly and operate under harsh conditions, making industrial application difficult. By contrast, biodegradation methods have attracted more attention given their advantages of high degradation efficiency, low cost, and secondary pollution-free (18). Screening of degradation microorganisms, degradation process optimization, and analysis of degradation products and kinetics are the main focuses in the biodegradation of SMX (19, 20). *Achromobacter* sp. JL9 (21), *Acinetobacte*r sp. (22), and *Pseudomonas psychrophila* HA-4 (23) have been identified as SMX degradation strains, achieving degradation efficiencies of 63.1%, 98.8%, and 34.3%, respectively. SMX can be degraded to aniline, 3-amino-5-methylisoxazole (3A5MI), 4-aminothiophenol, and sulfanilamide by *P. psychrophila* HA-4 (23). To further improve degradation, metabolic engineering can be used to create genetically editable environmentally friendly SMX degradation strains. However, the screened SMX degradation strains are difficult to engineer because of a lack of metabolic engineering tools and methods and a poor understanding of the degradation mechanisms and key genes.

**Fig 1:**
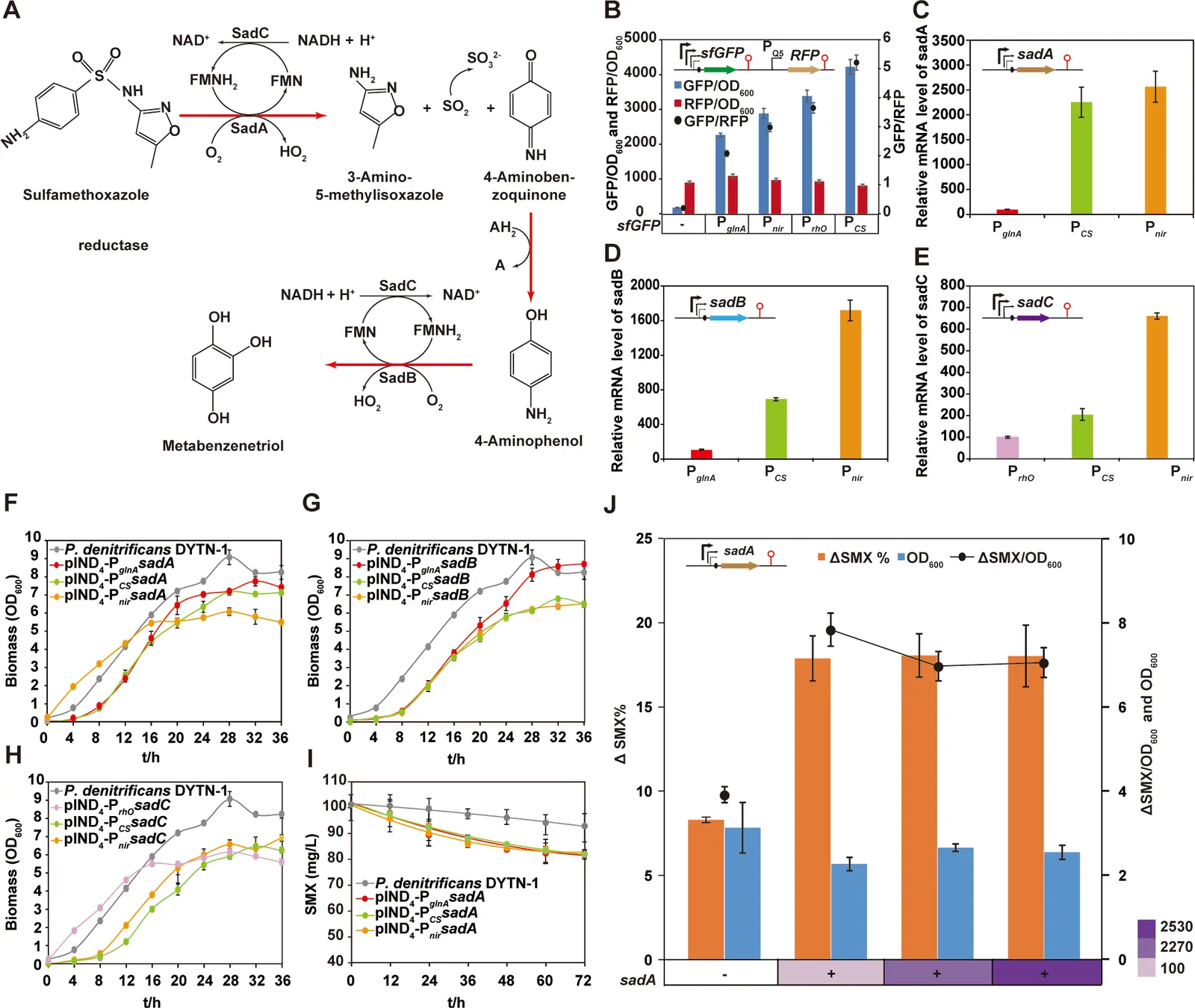
Gradient strength promoter characterization for the SMX degradation pathway. (A) The validated SMX degradation pathway. SadA and SadB are FMNH_2_-dependent monooxygenases; SadC is an FMN reductase that cooperates with SadA and SadB to degrade SMX. (B–E) Characterization of the promoter strength of P*_glnA_*, P*_nir_*, P*_rhO_*, and P*_CS_* by GFP/RFP fluorescence intensity (B) and mRNA levels of *sadA* (C), *sadB* (D), and *sadC* (E). (F–H) Growth curve of the wild-type and *sadA* (F), *sadB* (G), and *sadC* (H) expressing *P. denitrificans* DYTN-1 strains. (I) Degradation of 100 mg/L SMX by wild-type and *sadA*-overexpressing strains. (J) Degradation of SMX after 72 h cultivation of strains; 100, 2,270, and 2,530 represent the expression level of *sadA* controlled by P*_glnA_*, P*_CS_*, and P*_nir_*, respectively. Data represents the mean and standard deviation for three replicates.

Using genetically editable environmental microorganisms as chassis cells for metabolic engineering is a feasible approach to enhance SMX degradation and environmental bioremediation. To do so, a clear metabolic pathway for SMX degradation is essential. Recently, Ricken et al. isolated a strain of *Microbacterium* sp. BR1 that was able to use SMX as its sole carbon source (24). Using genomics, proteomics, and ^14^C-labeled metabolomics analyses, they identified a gene cluster involved in degradation that consisted of three functional genes, two monooxygenase genes (*sadA* and *sadB*), and a flavin mononucleotide (FMN) reductase gene (*sadC*). Through catalysis of SadA and SadB, SMX was degraded to 3A5MI, metabenzenetriol, and SO_2_ (24 – 26) (Fig. 1A). This discovery provided a clear genetic background and a potential for metabolic engineering to enhance the degradation of SMX.

To do so, a clear metabolic pathway for SMX degradation is essential. Recently, Ricken et al. isolated a strain of *Microbacterium sp*. BR1 that was able to use SMX as its sole carbon source (24). Using genomics, proteomics, and ^14^C-labeled metabolomics analyses, they identified a gene cluster involved in degradation that consisted of three functional genes, two monooxygenase genes (*sadA* and *sadB*), and an FMN reductase gene (*sadC*). Through catalysis of SadA and SadB, SMX was degraded to 3A5MI, metabenzenetriol, and SO_2_ (24 – 26) (Fig. 1A). This discovery provided a clear genetic background and a potential for metabolic engineering to enhance the degradation of SMX.

*P. denitrificans* is a genetically editable environmentally friendly strain with heterotrophic nitrifying and aerobic denitrifying properties (27), and is widely used in nitrogen removal of wastewater (28). Cloning and optimization of the SMX degradation pathway of *P. denitrificans* chassis would be a promising approach to reduce SMX pollution. In addition to SMX pollution, the problems caused by ammonia pollutants in the environment are becoming increasingly apparent. In recent years, the effect of SMX on nitrogen removal and microbial community of wastewater biological treatment systems has attracted attention. SMX affected the nitrogen removal process by influencing the abundance and diversity of the activated sludge microbial community (29). To address this, we constructed a dual-degradation *P. denitrificans* strain that could simultaneously remove SMX and ammonia nitrogen. In doing so, we systemically optimized the expression level and sources of components of the SMX degradation pathway by the characterized native promoters in this study. This optimized strain exhibited the ability to remove both SMX and ammonia nitrogen, and may be useful in environmental bioremediation.

## MATERIALS AND METHODS

### Strains, medium, and cultivation

*Escherichia coli* JM109 and *P. denitrificans* DYTN-1 (27, 30) were used for plasmid cloning and constitutive expression of SMX degradation genes. *E. coli* S17-1λpir was used as a helper strain for the conjugative transfer of *P. denitrificans* DYTN-1. Luria Bertani (LB) medium (Supporting information) was used for *E. coli* strain culture. M9 medium (Supporting information) was used to degrade SMX by *P. denitrificans* DYTN-1; 1 mL *P*. *denitrificans* DYTN-1 seed inoculates was cultured overnight, added to 50 mL M9 medium in 250 mL conical flasks, and cultured at 150 rpm at 30°C. Spectinomycin (50 µg/mL) and kanamycin (50 µg/mL) were added as required.

### Conjugation

*E. coli* S17-1λpir carrying a recombinant plasmid was used as a donor strain and *P. denitrificans* DYTN-1 as a recipient strain. Bacteria were inoculated in tubes containing 5 mL LB and cultured overnight at 37°C (*E. coli* S17-1λpir) or 30°C (*P. denitrificans* DYTN-1). The next day, a 3:10 ratio of *E. coli* S17-1λpir:*P. denitrificans* DYTN-1 was transferred to the same tube, mixed, and cultured for 30 min at 37°C. Then, the tube was shaken vigorously to disrupt the sexual fimbriae between donors and recipients, thus halting gene transfer. Finally, 0.1 mL conjugation liquid was coated on agar plates with the relevant antibiotic to generate positive clones.

### Plasmid construction

Plasmids and primers used in this study are listed in Tables S1 and S2. All DNA polymerases and DNA ligases were purchased from Takara (Dalian, China). pIND_4_-P*_rpsu_-sfGFP* is a pIND_4_ plasmid with P*_rpsu_* promoter controlled *sfGFP* (30). Using pIND_4_-P*_rpsu_-sfGFP* as a backbone, *sfGFP* was replaced by Azenta (Suzhou, China) synthesized *sadA*, *sadB*, and *sadC*, generating pIND_4_-P*_rpsu_-sadA*, pIND_4_-P*_rpsu_-sadB*, and pIND_4_-P*_rpsu_-sadC*. Then, using *P. denitrificans* DYTN-1 genomic DNA as template, the endogenous promoters, P*_glnA_*, P*_rhO_*, P*_CS_*, and P*_nir_* were amplified by the primer pairs of *glnA*-F/*glnA-GFP*-R, *rhO*-F/*rhO-GFP*-R, *CS*-F/*CS-GFP*-R, and *nir*-F/*nir-GFP*-R, to obtain the promoter fragments *glnA-GFP*, *rhO-GFP*, *CS-GFP*, and *nir-GFP*, respectively. These amplified promoter fragments were cloned upstream of *sfGFP* of pIND_4_-P*_rpsu_-sfGFP* to replace the promoter P*_rpsu_*, generating plasmids pIND_4_-P*_glnA_-sfGFP*, pIND_4_-P*_rhO_-sfGFP*, pIND_4_-P*_CS_-sfGFP*, and pIND_4_-P*_nir_-sfGFP*. In addition, the fragments of *RFP*, amplified by primer pairs *RFP*-F/*RFP*-R, were used to replace *sfGFP* of pIND_4_-P_Q5_-*sfGFP*, generating plasmid pIND_4_-P_Q5_-*RFP*. Then, the fragments of *cymR-RFP*, amplified from pIND_4_-P_Q5_-*RFP* by primer pairs *cymR*-F/*RFP*-R , were cloned into plasmids of pIND_4_-P*_glnA_-sfGFP*, pIND_4_-P*_rhO_-sfGFP*, pIND_4_-P*_CS_-sfGFP*, and pIND_4_-P*_nir_-sfGFP*, generating plasmids of pIND_4_-P*_glnA_-sfGFP*-P_Q5_-*RFP*, pIND_4_-P*_rhO_-sfGFP*-P_Q5_-*RFP*, pIND_4_-P*_CS_-sfGFP*-P_Q5_-*RFP*, and pIND_4_-P*_nir_-sfGFP*-P_Q5_-*RFP* (Fig. S1).

Similarly, the promoter fragments *glnA-A*, *glnA-B*, *rhO*-C, *CS-A*, *CS-B* ,*CS*-C, *nir-A*, *nir-B*, and *nir*-C were amplified by the primer pairs *glnA*-F/*glnA*-A-R, *glnA*-F/*glnA*-B-R, *rhO*-F/*rhO*-C-R, *CS*-F/*CS*-A-R, *CS*-F/*CS*-B-R, *CS*-F/*CS*-C-R, *nir*-F/*nir*-A-R, *nir*-F/*nir*-B-R, and *nir*-F/*nir*-C-R, respectively. These amplified promoter fragments were cloned upstream of *sadA*, *sadB*, and *sadC* of pIND_4_-P*_rpsu_-sadA*, pIND_4_-P*_rpsu_-sadB*, and pIND_4_-P*_rpsu_-sadC* to replace the promoter P*_rpsu_*, generating plasmids pIND_4_-P*_glnA_-sadA*, pIND_4_-P*_glnA_-sadB*, pIND_4_-P*_rhO_-sadC*, pIND_4_-P*_CS_-sadA*, pIND_4_-P*_CS_-sadB*, pIND_4_-P*_CS_-sadC*, pIND_4_-P*_nir_-sadA*, pIND_4_-P*_nir_-sadB*, and pIND_4_-P*_nir_-sadC* (Fig. S2).

The promoter-*sadB* DNA fragments of *glnAsadB*, *CSsadB*, and *nirsadB* were amplified from pIND_4_-P*_glnA_-sadB*, pIND_4_-P*_CS_-sadB*, and pIND_4_-P*_nir_-sadB* by primer pairs A-*glnAsadB*-F/B-R, A-*CSsadB*-F/B-R, and A-*nirsadB*-F/B-R, respectively. These amplified promoter-*sadB* DNA fragments were cloned downstream of *sadA* of pIND_4_-P*_glnA_-sadA*, pIND_4_-P*_CS_-sadA*, and pIND_4_-P*_nir_-sadA*, generating plasmids of pIND_4_-P*_glnA_-sadA*-P*_glnA_-sadB*, pIND_4_-P*_glnA_-sadA-*P*_CS_-sadB*, pIND_4_-P*_glnA_-sadA-*P*_nir_-sadB*, pIND_4_-P*_CS_-sadA-*P*_glnA_-sadB*, pIND_4_-P*_CS_-sadA-*P*_CS_-sadB*, pIND_4_-P*_CS_-sadA-*P_nir_ *-sadB*, pIND_4_-P*_nir_-sadA-*P*_glnA_-sadB*, pIND_4_-P*_nir_-sadA-*P*_CS_-sadB*, and pIND_4_-P*_nir_-sadA-*P*_nir_-sadB* (Fig. S3).

The promoter-*sadC* DNA fragments of *rhOsadC*, *CSsadC*, and *nirsadC* were amplified from pIND_4_-P*_rhO_-sadC*, pIND_4_-P*_CS_-sadC*, and pIND_4_-P*_nir_-sadC* with primer pairs B-*rhOsadC*-F/C-R, B-*CSsadC*-F/C-R, and B-*nirsadC*-F/C-R. These amplified promoter-*sadC* DNA fragments were cloned downstream of *sadB* of pIND_4_-P*_glnA_-sadA*-P*_glnA_-sadB* to generate three plasmids: pIAB_4_-P*_rhO_-sadC*, pIAB_4_-P*_CS_-sadC*, and pIAB_4_-P*_nir_-sadC* (Fig. S4). The gene coding sequences (CDSs) of *ssuE* and *rutF* were amplified from *E. coli* MG1655 with primer pairs *ssuE*-F*/ssuE*-R and *rutF*-F/*rutF*-R, respectively. The gene CDSs of *cogA*, *sutR*, and *psuK* were amplified from *C. glutamicum* ATCC 13032, *B. subtilis* SRCM102756, and *P. putida* KT2440 with primer pairs *cogA*-F/*cogA*-R, *sutR*-F/*sutR*-R, and *psuK*-F/*psuK*-R, respectively. These amplified genes were used to replace *sadC* of pIAB_4_-P*_rhO_-sadC*, pIAB_4_-P*_CS_-sadC*, and pIAB_4_-P*_nir_-sadC*, generating plasmids pIAB_4_ *-*P*_rhO_-ssuE*, pIAB_4_ *-*P*_rhO_-ssuE*, pIAB_4_ *-*P*_nir_-ssuE*, pIAB_4_ *-*P*_rhO_-rutF*, pIAB_4_ *-*P*_CS_-rutF*, pIAB_4_ *-*P*_nir_-rutF*, pIAB_4_ *-*P*_rhO_-cogA*, pIAB_4_ *-*P*_CS_-cogA*, pIAB_4_ *-*P*_nir_-cogA*, pIAB_4_ *-*P*_rhO_-sutR*, pIAB_4_ *-*P*_CS_-sutR*, pIAB_4_ *-*P*_nir_-sutR*, pIAB_4_ *-*P*_rhO_-psuK*, pIAB_4_ *-*P*_CS_-psuK*, and pIAB_4_ *-*P*_nir_-psuK*.

To measure the enzyme activity of SadC, SsuE, RutF, CogA, SutR, and PsuK, the CDSs of *sadC*, *ssuE*, *rutF*, *cogA*, *sutR*, and *psuK* were amplified with primer pairs H-*sadC*-F/H-*sadC*-R, H-*ssuE*-F/H-*ssuE*-R, H-*rutF*-F/H-*rutF*-R, H-*cogA*-F/H-*cogA*-R, H-*sutR*-F/H-*sutR*-R, and H-*psuK*-F/H-*psuK*-R, respectively. The amplified CDSs were cloned downstream of the 6 × His tag of pET-28a, generating plasmids pET-28a-*sadC*, pET-28a-*ssuE*, PET-28a-*rutF*, pET-28a-*cogA*, pET-28a-*sutR*, and pET-28a-*psuK* (Fig. S5).

### Characterization of promoters

First, *P. denitrificans* DYTN-1 strains carrying reported plasmids were cultured at 30°C and 150 rpm to OD_600_ of 0.4–0.6 ,then 50 µM cumate (4-isopropylbenzoic acid) was added. After 48 h of incubation, 20 µL of the activated cells was transferred to 96-well microtiter plates and diluted 10 times with phosphate-buffered saline (PBS) prior to fluorescence and OD_600_ measurements. Green fluorescence measurements were performed using a Biotek HT plate reader (Winooski, VT, USA) with excitation at 485 nm, emission at 528 nm, while red fluorescence measurements were with excitation at 588 nm, emission at 633 nm.

### Metabolite quantification

About 2 mL overnight cultured strains was inoculated in 50 mL modified M9 medium (Supplementary Notes) which contained 100 mg/L SMX, 1 g/L NO_3_^−^ or NH_4_^+^, then cultured at 30°C for 72 h. Culture supernatant was obtained by centrifugation at 15,000 × *g* for 5 min and diluted for metabolite quantification. Nitrate and ammonia nitrogen were determined using LH-NO_3_ Liquid and LH-NH_4_ Liquid (Lianhua, Shanghai, China) kits on a Biotek HT plate reader (BioTek, Winooski, VT, USA) with emission at 410 and 420 nm, respectively.

### High-performance liquid chromatography quantification

Equal volume of methanol and culturing broth was mixed and standing for 30 min. Through this process, both supernatant SMX and SMX contained within the bacteria were extracted from the mixture. Then mixture of supernatants was obtained by centrifugation at 15,000 × *g* for 5 min and filtered for high-performance liquid chromatography (HPLC) quantification. The concentration of SMX was analyzed by HPLC (Agilent 1260 II, Santa Clara, CA, USA) equipped with an EC-C18 column (4 µm, 4.6 × 150 mm^2^) at 35°C. SMX was measured under the detection of a VWD detector (Agilent, Santa Clara, CA, USA) at 265 nm. The mobile phase was a 1:3 ratio of acetonitrile and 0.2% acetic acid with a flow rate of 0.6 mL/min.

### Real-time quantitative fluorescence PCR (RT-qPCR)

Overnight cultured strains were inoculated in 25 mL M9 medium in a 1% inoculum. After incubation to logarithmic growth phase at 30°C and 150 rpm, the cells were collected for mRNA extraction and processed according to the manual with an RNA pure Bacteria Kit (CWbiotech, Beijing, China). Genomic DNA was then removed for reverse transcription and synthesis of cDNA using the HiFiScript gDNA Removal cDNA Synthesis Kit (CWbiotech). Real-time quantitative polymerase chain reaction(RT-qPCR) was performed using ultra SYBR mixtures (CWBiotech) on a CFX96 touch RT-qPCR detection system (Bio-Rad, Hercules, CA, USA). 16S rRNA was amplified using RT-16S-F/RT-16S-R primers and used as an internal reference. The primer pairs of RT-*sadA*-F/RT-*sadA*-R, RT-*sadB*-F/RT-*sadB*-R, RT-*sad*C-F/RT-*sadC*-R, RT-*ssuE*-F/RT-*ssuE*-R, RT-*rutF*-F/RT-*rutF*-R, RT-*cogA*-F/RT-*cogA*-R, RT-*sutR*-F/RT-*sutR*-R, and RT-*psuK*-F/RT-*psuK*-R were used to amplify *sadA*, *sadB*, *sadC*, *ssuE*, *rutF*, *cogA*, *sutR*, and *psuK*, respectively. Relative transcript levels of these genes were calculated using the 2^−∆∆CT^ method (31), with *P. denitrificans* DYTN-1 as the control.

### Purification and characterization of FMN reductases

First, pET-28a-*sadC*, pET-28a-*ssuE*, PET-28a-*rutF*, pET-28a-*cogA*, pET-28a-*sutR*, and pET-28a-*psuK* expressing *E. coli* BL21 (DE3) strains were cultured at 37°C and 200 rpm to OD_600_ of 0.8–0.9. Then, the temperature was shifted from 37°C to 20°C and 0.2 mM IPTG was added. After induction for 8 h, the cells were harvested by centrifugation at 10,000 × *g* for 10 min and resuspended in PBS standard buffer. Then, the cells were sonicated and the supernatant was harvested after centrifugation at 10,000 × *g* for 15 min. Finally, FMN reductases were purified by using affinity chromatography using Ni^2+^-NTA columns.

The activity of FMN reductases was detected by calculating the decrease of NADPH (NADH), using FMN as the substrate, at 30°C for 10 min. The reaction was measured on a Biotek HT plate reader (Winooski, VT, USA) with an absorbance of 340 nm. One unit of FMN reductase activity was defined as the amount of NADPH (NADH) decrease under 1 µmol enzyme per minute at 30°C.

## RESULTS

### Gradient strength promoter characterization to control expression of *sadABC*

As an important part of metabolic engineering, the promoter plays a major role in determining the gene expression level of engineered microorganisms (32, 33). Furthermore, Balakrishnan et al. revealed that the gene expression level was mainly controlled by the promoter and most mRNA has the similar translation level (34). In this regard, it is feasible to calculate and regulate the gene expression level by considering the promoter strength. To obtain gradient strength promoters to regulate the expression of SMX degradation genes, we selected and cloned the upstream intergenic region (35, 36) of *glnA* (glutamate-ammonia ligase), *nirI* (nitrite reductase), *rhO* (transcription termination factor Rho), and *cs* (citrate synthase) as promoter from the genome of *P. denitrificans* DYTN-1, named P*_glnA_*, P*_nir_*, P*_rhO_*, and P*_CS_*, respectively (Table S3). We constructed a reporter system (Fig. S1) containing the genes encoding the green fluorescent protein (GFP) and the red fluorescent protein (RFP) and normalized GFP signal against RFP signal (GFP/RFP) to calculate the ratio of strength of promoters (Fig. 1B). When using the pIND_4_-P_Q5_-*RFP* strain as control, there is no significant difference in the expression level of RFP among these strains, indicating a stable expression level of RFP (Fig. 1B). This stability makes RFP a reliable internal reference for quantifying the strength of promoters that control the expression of GFP. Finally, the strength ratio of P*_glnA_*:P*_nir_*:P*_rhO_*:P*_CS_* was 11:15:19:27. Thus, these promoters showed potential for use in regulating the expression of *sadABC*. Considering the promoter expression fluctuation that occurs when expressing different genes, the mRNA levels of *sadA*, *sadB*, and *sadC* under the control of the screened promoters were measured by RT-qPCR (Fig. 1C through E). Same promoter exhibited a significantly different mRNA transcription level when expressing different genes. The relative expression level of *sadA* was 100, 2,270, and 2,530 when using P*_glnA_*, P*_CS_*, and P*_nir_*, respectively, as promoters. The relative expression level of *sadB* was 100, 691, and 1,717 with P*_glnA_*, P*_CS_*, and P*_nir_*, respectively, as promoters. The relative expression level of *sadC* was 100, 205, and 661 with P*_rhO_*, P*_CS_*, and P*_nir_*, respectively, as promoters. These relative expression levels were used as the reference for further regulation of the SMX degradation pathway.

Compared with the wild-type strain (*P. denitrificans* DYTN-1), heterogenous expression of *sadA*, *sadB*, and *sadC* resulted in either slow growth or low final OD_600_ (Fig. 1F through H). More specifically, the higher expression level of *sadA* and *sadB* resulted in lower final OD_600_. However, the different expression levels of *sadC* results similar final OD_600_. The different expression levels of the SMX degradation genes would generate various degrees of cell growth inhibition which may be caused by the metabolic burden from plasmid replication and gene expression (37, 38). Hence, systemically optimizing the expression level of *sadA*, *sadB*, and *sadC* will be essential to construct an SMX degradation strain with desired performance.

To verify whether overexpression of *sadA* could provide SMX degradation for *P. denitrificans* DYTN-1, we cultivated *sadA*-overexpressing strains in a medium containing 100 mg/L SMX (Fig. S6). Compared with the wild-type strain, the SMX concentration of engineered strains obviously decreased (Fig. 1I). Considering detection error and photodegradation (39), it is reasonable to conclude that *P. denitrificans* DYTN-1 without exogenous gene expression had a 7.8% reduction in SMX concentration. Although the three promoters showed strength differences, SMX concentrations were degraded by approximately 18% in all three groups carrying the *sadA* gene in the degradation test, twofold higher than the wild-type strain. It was possible that without the help of SadB and SadC, the degradation of SMX was up to a bottleneck. In terms of degradation capacity per unit of biomass, the wild-type strain was at 3.1 mg/L/OD_600_, while *sadA*-overexpressing strains were approximately 7 mg/L/OD_600_ (Fig. 1J). This result coincided with the finding of Ricken that SadA attacked SMX and mediated the first degradation step of SMX (25, 26).

**Fig 2:**
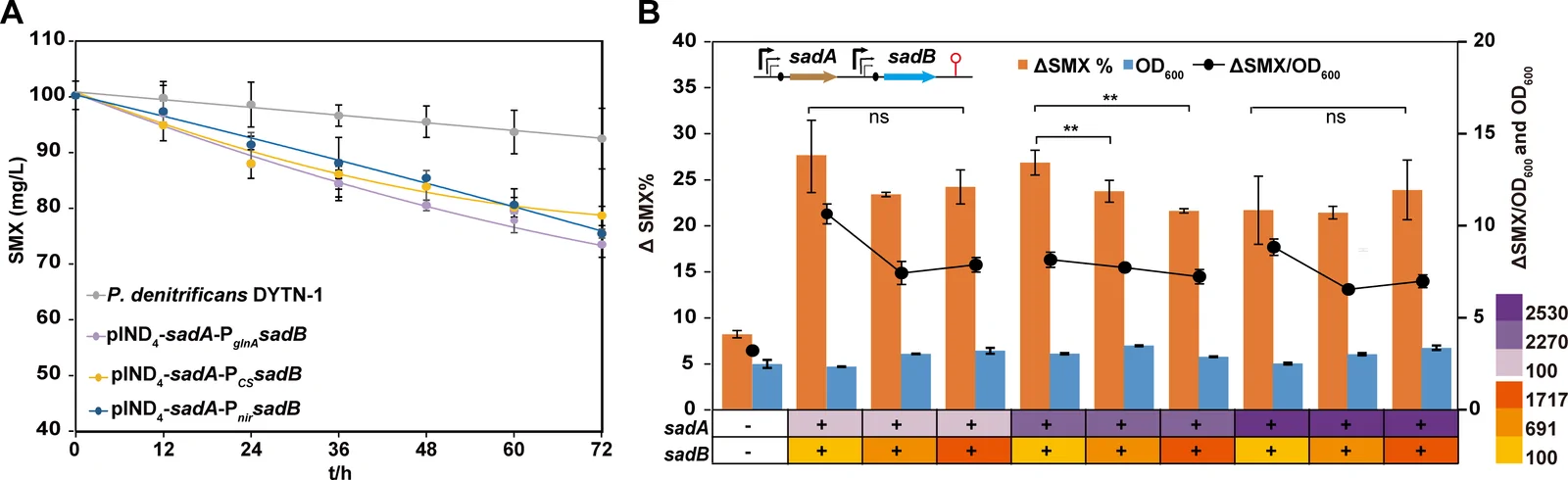
Degradation of 100 mg/L SMX by recombinant strains expressing *sadA* and *sadB* genes. (**A**) SMX degradation time course with recombinant strains. The expression of *sadA* was controlled by P*_CS_*. (**B**) Degradation of SMX after 72 h cultivation of *sadA-sadB* strains; 100, 2,270, and 2,530 represent the expression level of *sadA* controlled by P*_glnA_*, P*_CS_*, and P*_nir_*, respectively, while 100, 691, and 1,717 represent the expression level of *sadB* controlled by P*_glnA_*, P*_CS_*, and P*_nir_*, respectively; “ns” and “**” represent no significant difference and highly significant difference (*P* ≤ 0.01), respectively. Data represent the mean and standard deviation for three replicates.

In the degradation reaction, SadC, an FMN reductase, is essential to supply reduced flavin mononucleotide (26) for catalysis function of SadA and SadB. However, without the expression of SadC, *sadA-sadB* co-expression strain could also achieve SMX degradation (Fig. 2A). This is likely due to the natural existence of FMN reductases in *P. denitrificans* DYTN-1 (40, 41). Considering SMX degradation efficiency was similar in different *sadA* expression strains, we speculated that the expression of *sadA* reached a threshold when *sadB* and *sadC* were not co-expressed. Hence, co-expression and further optimization of the expression levels of *sadA*, *sadB*, and *sadC* will be essential for improving the degradation of SMX.

### Optimization of the expression level of *sadA* and *sadB* to improve degradation efficiency

The release of product can promote the use of the substrate (42, 43). Hence, combined expression of *sadB* with *sadA* may further improve the degradation efficiency of SMX. Due to the lack of terminator and intergenic region regulation elements in *P. denitrificans*, the expression level of *sadB* was regulated in a pseudo-operon structure by the above gradient strength promoters. The *sadA*-expressing strains degraded 100 mg/L SMX to 82 mg/L (Fig. 1I), while the best *sadA-sadB* co-expression strain degraded SMX to 72.3 mg/L (Fig. 2A). Thus, co-expression of the *sadB* gene was able to promote the degradation of SMX (Fig. S7). However, analyzing the *sadA-sadB* expressed strains, we found that the SMX degradation efficiency of most strains did not significantly improve with the optimization expression of *sadB* (Fig. 2B). Strain P*_glnA_-sadA*-P*_glnA_*-s*adB* had the highest degradation efficiency of 27.7%, while the degradation efficiencies of the other combinations ranged from 26.8% to 21%. Furthermore, when the *sadA* was expressed at a medium level, the degradation efficiency of its combinations with a low *sadB* expression level exhibited a significant improvement (Fig. 2B). While optimization the expression level of *sadB* in *sadA* low and high expressed strains had no significant difference in SMX degradation efficiency. This pointed that low expression of *sadA* was sufficient to maximize the degradation efficiency of SMX. Considering cell growth, the highest SMX degradation per OD_600_ was observed with the P*_glnA_-sadA*-P*_glnA_-sadB* strain at 10.6 mg/L/OD_600_; the other combination strains were in the range of 7–8 mg/L/OD_600_.

According to the aforementioned degradation efficiency of different combinations, we concluded that the co-expression of *sadA* and *sadB* at a low level could achieve their highest degradation ability, continuing to increase their expression level have no help in improving SMX degradation. We speculated that the shortage of FMNH_2_, a coenzyme of SadA and SadB, is likely the main factor that limited SMX degradation efficiency. Therefore, we compared and overexpressed different FMN reductases from different sources to improve the overall efficiency of the degradation process.

### FMN reductase optimization

A large number of flavin-dependent monooxygenases have been isolated and characterized (44, 45), including SadA and SadB, which require a flavin reductase, such as SadC, for electron transferring (25). In *E. coli*, the two-component alkanesulfonate monooxygenase system consists of an FMN reductase (SsuE) and a monooxygenase (SsuD) that together catalyze the oxidation of alkanesulfonate to the corresponding aldehyde and sulfite (46 – 48). It has been reported that *E. coli* grows with FMN reductase (RutF) as a coenzyme (49). We selected the other three FMN reductases from the NCBI database, derived from *Corynebacterium glutamicum* strain ATCC 13032 (CogA, gene ID: CP025533.1), *Bacillus subtilis* strain SRCM102756 (SutR, gene ID: CP028218.1), and *Pseudomonas putida* strain KT2440 (PsuK, gene ID: LT799039.1). Therefore, different sources of FMN reductase (*fmnR*)—*sadC*, *ssuE*, *rutF*, *cogA*, *sutR*, and *psuK*—were cloned and expressed into the pIND_4_-P*_glnA_-sadA*-P*_glnA_*-s*adB* strain, constructing *sadA-sadB-fmnR* strains to improve the supply of FMNH_2_.

**Fig 3:**
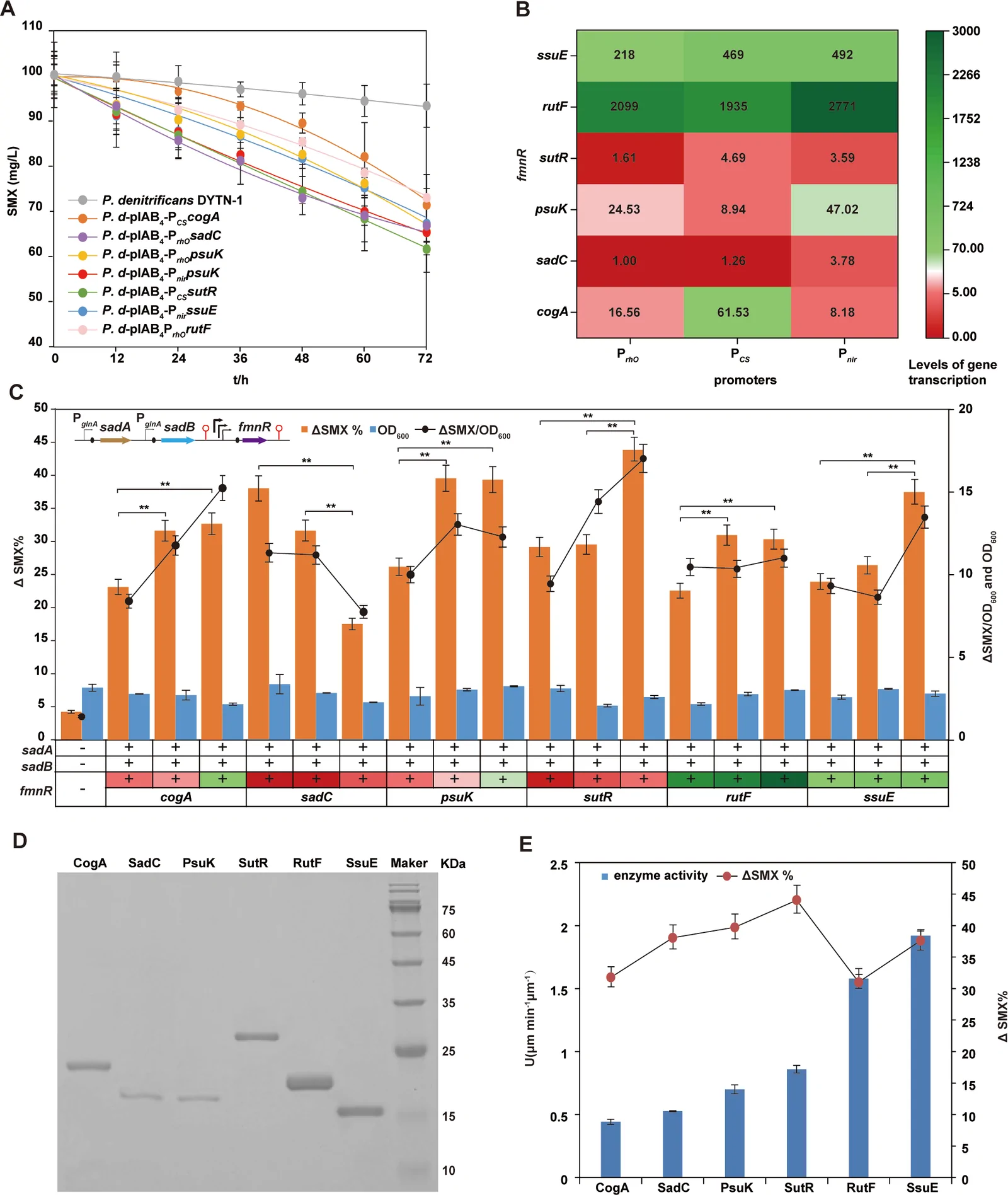
Optimization of FMN reductase source and expression level. (**A**) Time course of SMX degradation with recombinant strains. Different FMN reductase genes (*sadC*, *ssuE*, *rutF*, *cogA*, *sutR*, and *psuK*) were cloned into the pIND_4_-P*_glnA_-sadA*-P*_glnA_*-s*adB*, generating a series of pIAB_4_ plasmids. (**B**) Transcript levels of *fmnR* in *P. denitrificans* DYTN-1. *fmnR* represents the FMN reductase gene. (**C**) Degradation of SMX after 72 h cultivation of SadA, SadB, and FMN reductase co-expressing strains. “**” represents highly significant difference (*P* ≤ 0.01), more details were shown in Fig. S9. (**D**) SDS-PAGE analysis of the purified FMN reductases. (**E**) Enzyme activities and degradation efficiencies of the six FMN reductases. Data represent the mean and standard deviation for three replicates.

Overexpression of FMN reductase helped improve the degradation efficiency of SMX (Fig. 3A; Fig. S8). Moreover, we found that except for the SadC-overexpressing strain, the relationship between FMN reductase expression level (Fig. 3B) and SMX degradation efficiency showed the same trend among the FMN reductase-expressing strains (Fig. 3C; Fig. S10). More specifically, the low expression level of *sadC* resulted in higher SMX degradation efficiency, reaching 38.1% (11.3 mg/L/OD_600_). By contrast, in the other five FMN reductase-expressing strains, the degradation efficiency and degradation capacity per unit of biomass of the strains increased with the expression level of *fmnR* (Fig. S10). The highest degradation efficiency was achieved by the *sutR*-expressing strain, reaching 44% (17 mg/L/OD_600_). However, the effect of expressing different FMN reductase genes on improving degradation efficiency differed significantly. This may be due to the different catalytic activities of FMN reductases in *P. denitrificans* DYTN-1 from different sources. Therefore, we purified six FMN reductases (Fig. 3D) and determined the enzyme activities (Fig. 3E). The activities of CogA, SadC, PsuK SutR, RutF, and SsuE were 0.44, 0.53, 0.7, 0.86, 1.58, and 1.92 (U), respectively. For CogA, SadC, PsuK, and SutR overexpressing strains, degradation efficiency increased with increased enzyme activity. However, the high enzymatic activity and expression level of SsuE and RutF were found to be associated with lower SMX degradation. FMN reductase plays a crucial role in reducing FMN^2+^ to FMNH_2_, which serves as a co-factor in various cellular processes. However, high activity and expression of FMN reductase can lead to excessive accumulation of FMNH_2_, which may disrupt the metabolic balance and activate alternative pathways, thereby reducing SMX degradation. In summary, SMX degradation was not only highly dependent on enzyme activity but also relied on the property of regulatory elements.

### Dual degradation of SMX and nitrogen contaminants by engineered *P. denitrificans* DYTN-1

After the above metabolic engineering, *P. denitrificans* DYTN-1 obtained a novel SMX degradation function. Kassotaki et al. found that improvement of ammonia oxidation efficiency promoted the removal efficiency of SMX, and the process of ammonia oxidation and SMX metabolism were inextricably related (50). In this regard, SMX and nitrogen dual-contaminant co-degradation could be achieved in one engineered strain.

**Fig 4:**
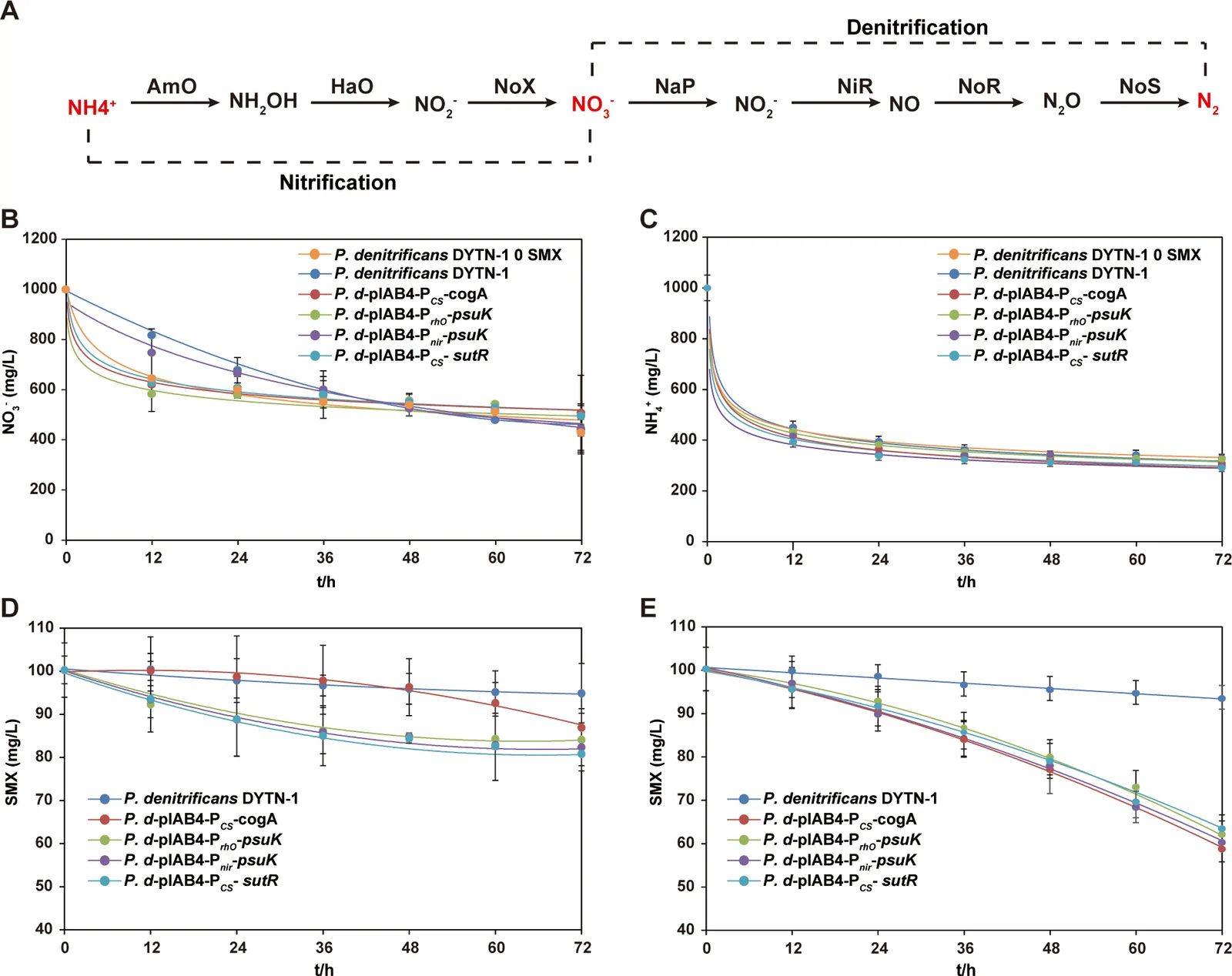
Co-degradation of SMX and nitrogen pollution by different engineered strains. (**A**) The process of denitrification and nitrification. AmO, ammonia monooxygenase; HaO, hydroxylamine oxidase; NoX, Nitrite oxidases; NaP, nitrate reductase; NiR, nitrite reductase; NoR, nitric oxide reductase; NoS, nitrous oxide reductase. (**B, C**) Changes in NO_3_^−^ (**B**) and NH _4_ ^+^ (**C**) during degradation. (**D, E**) Changes in SMX during degradation when NO_3_^−^ (**D**) or NH _4_ ^+^ (**E**) exist. *P. denitrificans* DYTN-10 SMX represents no SMX in the medium; 100 mg/L SMX was added to the medium of other strains. Data represent the mean and standard deviation for three replicates.

To investigate the impacts of SMX and SMX degradation genes on the nitrification and denitrification (Fig. 4A) of *P. denitrificans* DYTN-1, we tested and compared the wild-type and four recombinant strains for dual-degradation of SMX and nitrogen contaminants. After 12 h of treatment, the removal rate of NO_3_^−^ in the medium was 35.5% for the wild-type strain without the addition of SMX; addition of SMX decreased the removal rate to 18.4%. Thus, denitrification by the wild-type strain was inhibited by SMX in the early growth stage (Fig. 4B). The recombinant strains, expressing SMX degradation genes, mitigated the “SMX stress” with an average NO_3_^−^ removal rate of 33%. After 72 h, the removal rate of NO_3_^−^ for the wild-type strain was 57.2%, while the removal rates of *P. denitrificans* DYTN-1, *P. d-*pIAB_4_-P*_CS_-cogA*, *P. d-*pIAB_4_-P*_CS_-sutR*, *P. d-*pIAB_4_ *-*P*_rhO_-psuK*, and *P. d-*pIAB_4_ *-*P*_nir_-psuK* were 55.7%, 49.3%, 58.0%, 56.4%, and 55.7%, respectively. These results demonstrate that the expression of *sadAB-fmnR* did not affect the degradation of NO_3_^−^. By contrast, nitrification was not influenced by SMX or the expression of *sadAB-fmnR* (Fig. 4C). The NH_4_^+^ removal rates of the wild-type strain with or without the addition of SMX were 69.5% and 67.8%, respectively, while NH_4_^+^ removal rates for strains expressing SMX degradation genes were 68–71%. Hence, the expression of *sadAB-fmnR* did not have a negative impact on the removal of NH_4_^+^.

Furthermore, we measured the SMX and nitrogen contaminant co-degradation efficiency of the engineered strains. In the NH_4_-N containing condition, the tested strains could remove 71% of nitrogen and simultaneously degrade ~40% of SMX (Fig. 4D); SMX degradation efficiency of wild-type *P. dentirificans* DYTN-1 was only 6.3%. However, with only NO_3_-N present, the SMX degradation efficiency of *P. denitrificans* DYTN-1, *P. d-*pIAB_4_-P*_CS_-cogA*, pIAB_4_-P*_CS_-sutR*, *P. d-*pIAB_4_ *-*P*_rhO_-psuK*, and *P. d-*pIAB_4_ *-*P*_nir_-psuK* decreased significantly to 4.6%, 10.6%, 14.3%, 8.8%, and 12.6%, respectively (Fig. 4E). This result demonstrated that the existence and oxidation of NH_4_^+^ is essential for SMX degradation (50). Overall, we endowed the SMX degradation function to *P. denitrificans* DYTN-1, a superior nitrogen removal environment strain, exhibiting a potential ability to address both SMX and nitrogen pollution issues (51).

## DISCUSSION

Microbial degradation is currently recognized as one of the most effective methods to treat SMX contamination (52); the most important concern is improving the degradation efficiency of the degradating bacteria (53). To address this challenge, we cloned, overexpressed, and optimized an SMX degradation pathway in a genetic-editable environment-friendly *P. denitrificans* DYTN-1 strain, gradually improved the degradation efficiency of SMX by promoter optimization and isoenzyme replacement, and finally obtained strains that could co-degrade SMX and ammonia nitrogen. The constructed strain could degrade 44% of SMX and remove 71% of the ammonia nitrogen. To the best of our knowledge, this is the first report using metabolic engineering to enhance SMX degradation in a microorganism, providing a potential method for environment governance.

As an environmentally friendly strain with superior nitrification and denitrification capacity, *P. denitrificans* DYTN-1 is considered a good chassis candidate for metabolic engineering. Promoters are the important regulatory aspects of gene expression in metabolic engineering (32). Only two inducible promoters were available in *P. denitrificans*, i.e., P_A1/04/03_ and P_Q5_, induced by IPTG and 4-isopropylbenzoic acid, respectively. After IPTG induction, the expression level of CheY6 protein (reporter gene) from P_A1/04/03_ was approximately threefold higher than that of the uninduced strain (54). Kaczmarczyk et al. developed a cumate-inducible gene expression system that achieved a 4,700-fold induction change by promoter P_Q5_ (55). Although controlling the inducer concentration could regulate the gene expression level, it is still challenging to express multiple genes and regulate their expression in *P. denitrificans* DYTN-1. In this regard, screening and characterizing gradient constitutive promoters were necessary to regulate different genes at different expression levels. To do so, we mined four endogenous promoters from *P. denitrificans* DYTN-1. The promoters showed a gradient *sfGFP*, *sadA*, *sadB*, and *sadC* expression level with a range up to ~25.3-fold at the mRNA level. Hence, the characterized gradient strength promoters will be useful tools for metabolic engineering in *P. denitrificans* DYTN-1. Besides the promoters, other regulation tools of gene expression, including RBSs (56), terminators (57), and intergenic region regulation elements (58), are also essential to optimize gene expression in different operon structures. However, the lack of these tools in *P. denitrificans* hindered the systemic optimization of the SMX degradation pathway in a more effective operon structure. Hence, it is necessary to develop more precise aforementioned gene expression tools in *P. denitrificans* in the future to improve the degradation of SMX through metabolic engineering approaches.

Using different operon structures to regulate the expression of the same multi-gene pathway often generates different phenotypes (36). Lim et al. showed a mechanism that increasing transcription distance (the distance from the start of a gene to the end of the operon) results in increased expression, due to it provides more time for translation to occur during transcription. Thus the upstream genes would produce higher enzyme abundance compared with the downstream genes in operon structure, and the expression level of downstream genes was difficult to be regulated in operon structure (59). Therefore, in this study, we used the independent promoter to regulate the expression of multi-genes in pseudo-operon which would avoid the above-mentioned problem. In order to regulate the expression level of downstream genes in operon structure, Keasling group developed a tunable intergenic region toolbox in *E. coli*, could generate 100-fold difference in expression in operon structure (58). This strategy was of great significance to expand in *P. denitrificans* in the future.

Currently, *sadA*, *sadB*, and *sadC* are the only known SMX degradation pathway genes (26). However, these genes form an incomplete SMX degradation pathway; the degradation genes for the SadA and SadB degradation products 3A5MI and metabenzenetriol, respectively, are unknown. Furthermore, how SMX is transported into the cell is also unclear. In this regard, it is necessary to mine the genome of SMX degradating strains to identify the SMX transporter and the degradation genes of 3A5MI and metabenzenetriol. SMX degradation efficiency is highly dependent on enzyme activity, so screening for enzymes of high activity or protein engineering modification based on existing enzymes is a potential strategy to improve SMX degradation.

SMX has also been reported to have had an inhibitory effect on biological denitrification (60). Thus, the construction of SMX and nitrogen co-degradation strains will be useful for environmental bioremediation. The constructed *P. d-*pIAB_4_-P*_CS_-sutR* strain was a preliminary exploration of possible co-degradation of SMX and nitrogen. With the development of synthetic biology tools in *P. denitrificans* DYTN-1 and the increasing of genes identified in SMX degradation, metabolic engineering strategies could be applied to enhance SMX and nitrogen degradation performance. Furthermore, as the degradation of SMX and nitrogen contaminants often occurred by the activity of microflora in soil, water, and activated sludge environments, interactions among the engineered strain and native microflora are important to maintain stable and high degradation efficiency (61). In this regard, the construction of a microflora adaptive strain for enhancing SMX degradation stability could be a future focus. The biosafety of the engineered strains is also an important consideration. Auxotroph, conditional lethal, and nucleic acid removal regulation systems could be established to ensure the biosafety of engineered strains (62 – 64). Finally, we believe that with the development of synthetic biology in environmental biology, engineered strains with increased biosafety, high degradation efficiency, and environmental stability could be constructed for ecological remediation.

## SUPPLEMENTAL MATERIAL

Supplementary Table S1-S3 and Supplementary Fig. S1-S10 spectrum.00146-23-s0001.doc Supplementary Tables and Figures

ASM does not own the copyrights to Supplemental Material that may be linked to, or accessed through, an article. The authors have granted ASM a non-exclusive, world-wide license to publish the Supplemental Material files. Please contact the corresponding author directly for reuse.
