# Supplementary material for: Metabolic engineering of Paracoccus denitrificans for dual degradation of sulfamethoxazole and ammonia nitrogen
Source: Microbiol Spectr. 2023 Sep 21;11(5):e00146-23. doi: 10.1128/spectrum.00146-23 (PMC10581052; doi:10.1128/spectrum.00146-23)
Supplement: Supplementary Table S1-S3 and Supplementary Fig. S1-S10 — Supplementary Tables and Figures [file spectrum.00146-23-s0001.doc]

# Supplementary Information

**Metabolic engineering of *Paracoccus denitrificans* for dual-degradation of sulfamethoxazole and ammonia nitrogen**

Shenghu Zhou^1,2,#^, Rongrong Zhu^1,2,#^, Xiaoqian Niu^1,2^, Yunying Zhao^1,2*^, Yu Deng^1,2*^

^1^ National Engineering Research Center of Cereal Fermentation and Food Biomanufacturing, Jiangnan University, 1800 Lihu Road, Wuxi, Jiangsu 214122, China

^2^Jiangsu Provincial Research Center for Bioactive Product Processing Technology, Jiangnan University, 1800 Lihu Road, Wuxi, Jiangsu 214122, China

Mailing address: National Engineering Laboratory for Cereal Fermentation Technology, 1800 Lihu Road, Wuxi, Jiangsu 214122, China

Phone: +86-510-85329031, Fax: +86-510-85918312

^#^ The authors contributed equally.

^*^Corresponding authors:

Yu Deng, Email: dengyu@jiangnan.edu.cn

Yunying Zhao, Email: yunyingzhao@jiangnan.edu.cn

# Table of Contents

[Supplementary Table 1 Strains and plasmids used in this study 3](#__RefHeading___Toc134051794)

[Supplementary Table 2 Primers used in this study 12](#__RefHeading___Toc134051795)

[Supplementary Table 3 DNA sequences of promoters, genes, and pIND4-P*_glnA_*-*sfGFP*-P_Q5_-*RFP* used in this study 16](#__RefHeading___Toc134051796)

[Supplementary Figure 1. The plasmid schematic diagrams and construction processes of plasmids that used to measure strength of promoters. 32](#__RefHeading___Toc134051797)

[Supplementary Figure 2. The plasmid schematic diagrams and construction processes of plasmids that used to measure strength of promoters. 33](#__RefHeading___Toc134051798)

[Supplementary Figure 3. The plasmid schematic diagrams and construction processes of *sadA* and *sadB* combinatorial plasmids under the controlling of different promoters. 34](#__RefHeading___Toc134051799)

[Supplementary Figure 4. The plasmid schematic diagrams and construction processes of plasmids harboring gene *sadA*-sadB-*fmnR*. 37](#__RefHeading___Toc134051800)

[Supplementary Figure 5. The plasmid schematic diagrams of plasmids with different FMN reductase genes. 38](#__RefHeading___Toc134051801)

[Supplementary Figure 6. Time course of OD_600_ (A), ΔSMX (B), and ΔSMX/OD_600_ (C) with wild-type and *sadA* strains. 39](#__RefHeading___Toc134051802)

[Supplementary Figure 7. Time course of OD_600_ (A), ΔSMX (B), and ΔSMX/OD_600_ (C) with wild-type and *sadA*-*sadB* strains. 40](#__RefHeading___Toc134051803)

[Supplementary Figure 8. Time course of OD_600_ (A), ΔSMX (B), and ΔSMX/OD_600_ (C) with wild-type and *sadA*-*sadB-fmnR* strains. 41](#__RefHeading___Toc134051804)

[Supplementary Figure 9. The Statistical analysis for degradation efficiency of *sadA*-*sadB-fmnR* strains. 42](#__RefHeading___Toc134051805)

[Supplementary Figure 10. The scatter plot of degradation efficiencies with different levels of transcription in *cogA* (A), *sadC*(B), *psuK*(C), *stuR*(D), *rutF*(E), *ssuE*(F) strains. 43](#__RefHeading___Toc134051806)

[Supplementary Notes 44](#__RefHeading___Toc134051807)

# Supplementary Table 1 Strains and plasmids used in this study

| **Name** | **Properties** | **Sources** |
| --- | --- | --- |
| **Strains** |  |  |
| *P. denitrificans* DYTN-1 | Wild type, for gene expression | Lab preserved |
| *E. coli* JM109 | Wild type, for plasmid construction | Lab preserved |
| *E. coli* S17-1λpir | Wild type, plasmid transfer helper strain | Lab preserved |
| *E. coli* BL21 | Wild type, for gene expression | Lab preserved |
| *E. coli* MG1655 | Wild type, genome carrying *ssuE* and *rutF* | Lab preserved |
| *C. glutamicum* strain ATCC 13032 | Wild type, genome carrying *cogA* | Lab preserved |
| *B. subtilis* strain SRCM102756 | Wild type, genome carrying *sutR* | Lab preserved |
| *P. putida* strain KT2440 | Wild type, genome carrying *psuK* | Lab preserved |
| pIND_4_-P_Q5_-*RFP* | *P. denitrificans* DYTN-1 carrying pIND_4_-P_Q5_-*RFP* | This study |
| pIND_4_-P*_glnA_*-*sfGFP*-P_Q5_-*RFP* | *P. denitrificans* DYTN-1 carrying pIND_4_-P*_glnA_*- *sfGFP*-P_Q5_-*RFP* | This study |
| pIND_4_-P*_rhO_*-*sfGFP*-P_Q5_-*RFP* | *P. denitrificans* DYTN-1 carrying pIND_4_-P*_rhO_*- *sfGFP*-P_Q5_-*RFP* | This study |
| pIND_4_-P*_CS_*-*sfGFP*-P_Q5_-  *RFP* | *P. denitrificans* DYTN-1 carrying pIND_4_-P*_CS_*- *sfGFP*-P_Q5_-*RFP* | This study |
| pIND_4_-P*_nir_*-*sfGFP*-P_Q5_-  *RFP* | *P. denitrificans* DYTN-1 carrying pIND_4_-P*_nir_*- *sfGFP*-P_Q5_-*RFP* | This study |
| pIND_4_-P*_rpsu_*-*sadA* | *P. denitrificans* DYTN-1 carrying pIND_4_-P*_rpsu_*-*sadA* | This study |
| pIND_4_-P*_rpsu_*-*sadB* | *P. denitrificans* DYTN-1 carrying pIND_4_-P*_rpsu_*-*sadB* | This study |
| pIND_4_-P*_rpsu_*-*sadC* | *P. denitrificans* DYTN-1 carrying pIND_4_-P*_rpsu_*-*sadC* | This study |
| pIND_4_-P*_glnA_*-*sadA* | *P. denitrificans* DYTN-1 carrying pIND_4_-P*_glnA_*-*sadA* | This study |
| pIND_4_-P*_glnA_*-*sadB* | *P. denitrificans* DYTN-1 carrying pIND_4_-P*_glnA_*-*sadB* | This study |
| pIND_4_-P*_rhO_*-*sadC* | *P. denitrificans* DYTN-1 carrying pIND_4_-P*_rhO_*-*sadC* | This study |
| pIND_4_-P*_CS_*-*sadA* | *P. denitrificans* DYTN-1 carrying pIND_4_-P*_CS_*-*sadA* | This study |
| pIND_4_-P*_CS_*-*sadB* | *P. denitrificans* DYTN-1 carrying pIND_4_-P*_CS_*-*sadB* | This study |
| pIND_4_-P*_CS_*-*sadC* | *P. denitrificans* DYTN-1 carrying pIND_4_-P*_CS_*-*sadC* | This study |
| pIND_4_-P*_nir_*-*sadA* | *P. denitrificans* DYTN-1 carrying pIND_4_-P*_nir_*-*sadA* | This study |
| pIND_4_-P*_nir_*-*sadB* | *P. denitrificans* DYTN-1 carrying pIND_4_-P*_nir_*-*sadB* | This study |
| pIND_4_-P*_nir_*-*sadC* | *P. denitrificans* DYTN-1 carrying pIND_4_-P*_nir_*-*sadC* | This study |
| pIND_4_-P*_nir_*- *sfGFP* | *P. denitrificans* DYTN-1 carrying pIND_4_-P*_nir_*- *sfGFP* | This study |
| pIND_4_-P*_nir_*-*sadA* | *P. denitrificans* DYTN-1 carrying pIND_4_-P*_nir_*-*sadA* | This study |
| pIND_4_-P*_nir_*-*sadB* | *P. denitrificans* DYTN-1 carrying pIND_4_-P*_nir_*-*sadB* | This study |
| pIND_4_-P*_nir_*-*sadC* | *P. denitrificans* DYTN-1 carrying pIND_4_-P*_nir_*-*sadC* | This study |
| pIND_4_-P*_glnA_-sadA-*P*_glnA_-*  *sadB* | *P. denitrificans* DYTN-1 carrying pIND_4_-P*_glnA_-sadA-*P*_glnA_-sadB* | This study |
| pIND_4_-P*_glnA_-sadA-*P*_CS_-*  *sadB* | *P. denitrificans* DYTN-1 carrying pIND_4_-P*_glnA_-sadA-*P*_CS_-sadB* | This study |
| pIND_4_-P*_glnA_-sadA-*P*_nir_-*  *sadB* | *P. denitrificans* DYTN-1 carrying pIND_4_-P*_glnA_-sadA-*P*_nir_-sadB* | This study |
| pIND_4_-P*_CS_-sadA-*P*_glnA_-*  *sadB* | *P. denitrificans* DYTN-1 carrying pIND_4_-P*_CS_-sadA-*P*_glnA_-sadB* | This study |
| pIND_4_-P*_CS_-sadA-*P*_CS_-*  *sadB* | *P. denitrificans* DYTN-1 carrying pIND_4_-P*_CS_-sadA-*P*_CS_-sadB* | This study |
| pIND_4_-P*_CS_-sadA-*P*_nir_-*  *sadB* | *P. denitrificans* DYTN-1 carrying pIND_4_-P*_CS_-sadA-*P*_nir_-sadB* | This study |
| pIND_4_-P*_nir_-sadA-*P*_glnA_-*  *sadB* | *P. denitrificans* DYTN-1 carrying pIND_4_-P*_nir_-sadA-*P*_glnA_-sadB* | This study |
| pIND_4_-P*_nir_-sadA-*P*_CS_-*  *sadB* | *P. denitrificans* DYTN-1 carrying pIND_4_-P*_nir_-sadA-*P*_CS_-sadB* | This study |
| pIND_4_-P*_nir_-sadA-*P*_nir_-*  *sadB* | *P. denitrificans* DYTN-1 carrying pIND_4_-P*_nir_-sadA-*P*_nir_-sadB* | This study |
| *P. d*-pIAB_4_*-*P*_rhO_-sadC* | *P. denitrificans* DYTN-1 carrying pIAB_4_*-*P*_rhO_-sadC* | This study |
| *P. d*-pIAB_4_*-*P*_CS_-sadC* | *P. denitrificans* DYTN-1 carrying pIAB_4_*-*P*_CS_-sadC* | This study |
| *P. d*-pIAB_4_*-*P*_nir_-sadC* | *P. denitrificans* DYTN-1 carrying pIAB_4_*-*P*_nir_-sadC* | This study |
| *P. d*-pIAB_4_*-*P*_rhO_-ssuE* | *P. denitrificans* DYTN-1 carrying pIAB_4_*-*P*_rhO_-ssuE* | This study |
| *P. d*-pIAB_4_*-*P*_CS_-ssuE* | *P. denitrificans* DYTN-1 carrying pIAB_4_*-*P*_CS_-ssuE* | This study |
| *P. d*-pIAB_4_*-*P*_nir_-ssuE* | *P. denitrificans* DYTN-1 carrying pIAB_4_*-*P*_nir_-ssuE* | This study |
| *P. d*-pIAB_4_*-*P*_rhO_-rutF* | *P. denitrificans* DYTN-1 carrying pIAB_4_*-*P*_rhO_-rutF* | This study |
| *P. d*-pIAB_4_*-*P*_CS_-rutF* | *P. denitrificans* DYTN-1 carrying pIAB_4_*-*P*_CS_-rutF* | This study |
| *P. d*-pIAB_4_*-*P*_nir_-rutF* | *P. denitrificans* DYTN-1 carrying pIAB_4_*-*P*_nir_-rutF* | This study |
| *P. d*-pIAB_4_*-*P*_rhO_-cogA* | *P. denitrificans* DYTN-1 carrying pIAB_4_*-*P*_rhO_-cogA* | This study |
| *P. d*-pIAB_4_*-*P*_CS_-cogA* | *P. denitrificans* DYTN-1 carrying pIAB_4_*-*P*_CS_-cogA* | This study |
| *P. d*-pIAB_4_*-*P*_nir_-cogA* | *P. denitrificans* DYTN-1 carrying pIAB_4_*-*P*_nir_-cogA* | This study |
| *P. d*-pIAB_4_*-*P*_rhO_-sutR* | *P. denitrificans* DYTN-1 carrying pIAB_4_*-*P*_rhO_-sutR* | This study |
| *P. d*-pIAB_4_*-*P*_CS_-sutR* | *P. denitrificans* DYTN-1 carrying pIAB_4_*-*P*_CS_-sutR* | This study |
| *P. d*-pIAB_4_*-*P*_nir_-sutR* | *P. denitrificans* DYTN-1 carrying pIAB_4_*-*P*_nir_-sutR* | This study |
| *P. d*-pIAB_4_*-*P*_rhO_-psuK* | *P. denitrificans* DYTN-1 carrying pIAB_4_*-*P*_rhO_-psuK* | This study |
| *P. d*-pIAB_4_*-*P*_CS_-psuK* | *P. denitrificans* DYTN-1 carrying pIAB_4_*-*P*_CS_-psuK* | This study |
| *P. d*-pIAB_4_*-*P*_nir_-psuK* | *P. denitrificans* DYTN-1 carrying pIAB_4_*-*P*_nir_-psuK* | This study |
| BL21-pET-28a-*sadC* | *E. coli* BL21 carrying pET-28a-*sadC* | This study |
| BL21-pET-28a-*ssuE* | *E. coli* BL21 carrying pET-28a-*ssuE* | This study |
| BL21-pET-28a-*rutF* | *E. coli* BL21 carrying pET-28a-*rutF* | This study |
| BL21-pET-28a-*cogA* | *E. coli* BL21 carrying pET-28a-*cogA* | This study |
| BL21-pET-28a-*sutR* | *E. coli* BL21 carrying pET-28a-*sutR* | This study |
| BL21-PET-28a-*psuK* | *E. coli* BL21 carrying PET-28a-*psuK* | This study |
| **Plasmids** |  |  |
| pIND_4_ | colE ori, repA, Kan^R^ | Lab preserved |
| pIND_4_-P*_rpsu_*-*sfGFP* | pIND_4_ backbone, P*_rpsu_* controlling the expression of *sfGFP*, Kan^R^ | Lab preserved |
| pIND_4_-P_Q5_-*sfGFP* | pIND_4_ backbone, *sfGFP*, Kan^R^ | Lab preserved |
| pET-28a | F1 ori, lacI, T7, 6×His tag, Kan^R^ | Lab preserved |
| pIND_4_-P*_rpsu_*-*sadA* | pIND_4_ backbone, *sadA*, Kan^R^ | Synthetic by Azenta |
| pIND_4_-P*_rpsu_*-*sadB* | pIND_4_ backbone, *sadB*, Kan^R^ | Synthetic by Azenta |
| pIND_4_-P*_rpsu_*-*sadC* | pIND_4_ backbone, *sadC*, Kan^R^ | Synthetic by Azenta |
| pIND_4_-P*_glnA_*-*sfGFP* | pIND_4_ backbone, P*_glnA_* controlling the expression of *sfGFP*, Kan^R^ | This study |
| pIND_4_-P*_rhO_*-*sfGFP* | pIND_4_ backbone, P*_rhO_* controlling the expression of *sfGFP*, Kan^R^ | This study |
| pIND_4_-P*_CS_*-*sfGFP* | pIND_4_ backbone, P*_CS_* controlling the expression of *sfGFP*, Kan^R^ | This study |
| pIND_4_-P*_nir_*-*sfGFP* | pIND_4_ backbone, P*_nir_* controlling the expression of *sfGFP*, Kan^R^ | This study |
| pIND_4_-P_Q5_-*RFP* | pIND_4_ backbone, P_Q5_ controlling the expression of *RFP*, Kan^R^ | This study |
| pIND_4_-P*_glnA_*-*sfGFP*-P_Q5_-*RFP* | pIND_4_ backbone, P*_glnA_* controlling the expression of *sfGFP*, P_Q5_ controlling the expression of *RFP*, Kan^R^ | This study |
| pIND_4_-P*_rhO_*-*sfGFP*-P_Q5_-*RFP* | pIND_4_ backbone, P*_rhO_* controlling the expression of *sfGFP*, P_Q5_ controlling the expression of *RFP*, Kan^R^ | This study |
| pIND_4_-P*_CS_*-*sfGFP*-P_Q5_-  *RFP* | pIND_4_ backbone, P*_CS_* controlling the expression of *sfGFP*, P_Q5_ controlling the expression of *RFP*, Kan^R^ | This study |
| pIND_4_-P*_nir_*-*sfGFP*-P_Q5_-  *RFP* | pIND_4_ backbone, P*_nir_* controlling the expression of *sfGFP*, P_Q5_ controlling the expression of *RFP*, Kan^R^ | This study |
| pIND_4_-P*_glnA_*-*sadA* | pIND_4_ backbone, P*_glnA_* controlling the expression of *sadA*, Kan^R^ | This study |
| pIND_4_-P*_glnA_*-*sadB* | pIND_4_ backbone, P*_glnA_* controlling the expression of *sadB*, Kan^R^ | This study |
| pIND_4_-P*_rhO_*-*sadC* | pIND_4_ backbone, P*_rhO_* controlling the expression of *sadC*, Kan^R^ | This study |
| pIND_4_-P*_CS_*-*sadA* | pIND_4_ backbone, P*_CS_* controlling the expression of *sadA*, Kan^R^ | This study |
| pIND_4_-P*_CS_*-*sadB* | pIND_4_ backbone, P*_CS_* controlling the expression of *sadB*, Kan^R^ | This study |
| pIND_4_-P*_CS_*-*sadC* | pIND_4_ backbone, P*_CS_* controlling the expression of *sadC*, Kan^R^ | This study |
| pIND_4_-P*_nir_*-*sadA* | pIND_4_ backbone, P*_nir_* controlling the expression of *sadA*, Kan^R^ | This study |
| pIND_4_-P*_nir_*-*sadB* | pIND_4_ backbone, P*_nir_* controlling the expression of *sadB*, Kan^R^ | This study |
| pIND_4_-P*_nir_*-*sadC* | pIND_4_ backbone, P*_nir_* controlling the expression of *sadC*, Kan^R^ | This study |
| pIND_4_-P*_glnA_-sadA-*P*_glnA_-*  *sadB* | pIND_4_ backbone, P*_glnA_* controlling the expression of *sadA*, P*_glnA_* controlling the expression of *sadB*, Kan^R^ | This study |
| pIND_4_-P*_glnA_-sadA-*P*_CS_-*  *sadB* | pIND_4_ backbone, P*_glnA_* controlling the expression of *sadA*, P*_CS_* controlling the expression of *sadB*, Kan^R^ | This study |
| pIND_4_-P*_glnA_-sadA-*P*_nir_-*  *sadB* | pIND_4_ backbone, P*_glnA_* controlling the expression of *sadA*, P*_nir_* controlling the expression of *sadB*, Kan^R^ | This study |
| pIND_4_-P*_CS_-sadA-*P*_glnA_-*  *sadB* | pIND_4_ backbone, P*_CS_* controlling the expression of *sadA*, P*_glnA_* controlling the expression of *sadB*, Kan^R^ | This study |
| pIND_4_-P*_CS_-sadA-*P*_CS_-*  *sadB* | pIND_4_ backbone, P*_CS_* controlling the expression of *sadA*, P*_CS_* controlling the expression of *sadB*, Kan^R^ | This study |
| pIND_4_-P*_CS_-sadA-*P*_nir_-*  *sadB* | pIND_4_ backbone, P*_CS_* controlling the expression of *sadA*, P*_nir_* controlling the expression of *sadB*, Kan^R^ | This study |
| pIND_4_-P*_nir_-sadA-*P*_glnA_-*  *sadB* | pIND_4_ backbone, P*_nir_* controlling the expression of *sadA*, P*_glnA_* controlling the expression of *sadB*, Kan^R^ | This study |
| pIND_4_-P*_nir_-sadA-*P*_CS_-*  *sadB* | pIND_4_ backbone, P*_nir_* controlling the expression of *sadA*, P*_CS_* controlling the expression of *sadB*, Kan^R^ | This study |
| pIND_4_-P*_nir_-sadA-*P*_nir_-*  *sadB* | pIND_4_ backbone, P*_nir_* controlling the expression of *sadA*, P*_nir_* controlling the expression of *sadB*, Kan^R^ | This study |
| pIAB_4_*-*P*_rhO_-sadC* | pIND_4_-P*_glnA_-sadA-*P*_glnA_-sadB* backbone, P*_rhO_* controlling the expression of *sadC* backbone, Kan^R^ | This study |
| pIAB_4_*-*P*_CS_-sadC* | pIND_4_-P*_glnA_-sadA-*P*_glnA_-sadB* backbone, P*_CS_* controlling the expression of *sadC* backbone, Kan^R^ | This study |
| pIAB_4_*-*P*_nir_-sadC* | pIND_4_-P*_glnA_-sadA-*P*_glnA_-sadB* backbone, P*_nir_* controlling the expression of *sadC*, Kan^R^ | This study |
| pIAB_4_*-*P*_rhO_-ssuE* | pIND_4_-P*_glnA_-sadA-*P*_glnA_-sadB* backbone, P*_rhO_* controlling the expression of *ssuE*, Kan^R^ | This study |
| pIAB_4_*-*P*_CS_-ssuE* | pIND_4_-P*_glnA_-sadA-*P*_glnA_-sadB* backbone, P*_CS_* controlling the expression of *ssuE*, Kan^R^ | This study |
| pIAB_4_*-*P*_nir_-ssuE* | pIND_4_-P*_glnA_-sadA-*P*_glnA_-sadB* backbone, P*_nir_* controlling the expression of *ssuE*, Kan^R^ | This study |
| pIAB_4_*-*P*_rhO_-rutF* | pIND_4_-P*_glnA_-sadA-*P*_glnA_-sadB* backbone, P*_rhO_* controlling the expression of *rutF*, Kan^R^ | This study |
| pIAB_4_*-*P*_CS_-rutF* | pIND_4_-P*_glnA_-sadA-*P*_glnA_-sadB* backbone, P*_CS_* controlling the expression of *rutF*, Kan^R^ | This study |
| pIAB_4_*-*P*_nir_-rutF* | pIND_4_-P*_glnA_-sadA-*P*_glnA_-sadB* backbone, P*_nir_* controlling the expression of *rutF*, Kan^R^ | This study |
| pIAB_4_*-*P*_rhO_-cogA* | pIND_4_-P*_glnA_-sadA-*P*_glnA_-sadB* backbone, P*_rhO_* controlling the expression of *cogA*, Kan^R^ | This study |
| pIAB_4_*-*P*_CS_-cogA* | pIND_4_-P*_glnA_-sadA-*P*_glnA_-sadB* backbone, P*_CS_* controlling the expression of *cogA*, Kan^R^ | This study |
| pIAB_4_*-*P*_nir_-cogA* | pIND_4_-P*_glnA_-sadA-*P*_glnA_-sadB* backbone, P*_nir_* controlling the expression of *cogA*, Kan^R^ | This study |
| pIAB_4_*-*P*_rhO_-sutR* | pIND_4_-P*_glnA_-sadA-*P*_glnA_-sadB* backbone, P*_rhO_* controlling the expression of *sutR*, Kan^R^ | This study |
| pIAB_4_*-*P*_CS_-sutR* | pIND_4_-P*_glnA_-sadA-*P*_glnA_-sadB* backbone, P*_CS_* controlling the expression of *sutR*, Kan^R^ | This study |
| pIAB_4_*-*P*_nir_-sutR* | pIND_4_-P*_glnA_-sadA-*P*_glnA_-sadB* backbone, P*_nir_* controlling the expression of *sutR*, Kan^R^ | This study |
| pIAB_4_*-*P*_rhO_-psuK* | pIND_4_-P*_glnA_-sadA-*P*_glnA_-sadB* backbone, P*_rhO_* controlling the expression of *psuK*, Kan^R^ | This study |
| pIAB_4_*-*P*_CS_-psuK* | pIND_4_-P*_glnA_-sadA-*P*_glnA_-sadB* backbone, P*_CS_* controlling the expression of *psuK*, Kan^R^ | This study |
| pIAB_4_*-*P*_nir_-psuK* | pIND_4_-P*_glnA_-sadA-*P*_glnA_-sadB* backbone, P*_nir_* controlling the expression of *psuK*, Kan^R^ | This study |
| pET-28a-*sadC* | pET-28a backbone, T7 controlling the expression of *sadC*, Kan^R^ | This study |
| pET-28a-*ssuE* | pET-28a backbone, T7 controlling the expression of *ssuE*, Kan^R^ | This study |
| pET-28a-*rutF* | pET-28a backbone, T7 controlling the expression of *rutF*, Kan^R^ | This study |
| pET-28a-*cogA* | pET-28a backbone, T7 controlling the expression of *cogA*, Kan^R^ | This study |
| pET-28a-*sutR* | pET-28a backbone, T7 controlling the expression of *sutR*, Kan^R^ | This study |
| PET-28a-*psuK* | pET-28a backbone, T7 controlling the expression of *psuK*, Kan^R^ | This study |

# Supplementary Table 2 Primers used in this study

| **Primer** | **Sequence** (**5' to 3'**)* | **Restriction sites** |
| --- | --- | --- |
| ***glnA*-F** | CATTAATTGCGTTGCGCTCGGCGACGGCAAGATCTTC |  |
| ***glnA*-*GFP*-R** | CACCTATACGCACCATTGGGATCCTTCCTCCATCAGTC |  |
| ***rhO*-F** | CATTAATTGCGTTGCGCAAGAATTGACTCGCGCCCGG |  |
| ***rhO*-*GFP*-R** | CACCTATACGCACCATGATCGGTCCGCGCAGAAAACTG |  |
| ***CS*-F** | CATTAATTGCGTTGCGCGGCCGCCAAGCAGATAGGC |  |
| ***CS*-*GFP*-R** | CACCTATACGCACCATTCCCAGTTCCTTTCACCTGTCTCC |  |
| ***nir*-F** | CATTAATTGCGTTGCGCGGGCGGCCTGTTCCTGTG |  |
| ***nir*-*GFP*-R** | CACCTATACGCACCATCCGCGTCCCAGGGTTCGT |  |
| ***RFP*-F** | CATGGCATGTATGGTGAGCGAGCTGATTAAGGAG |  |
| ***RFP-*R** | GAAGCCATCACTCATCTGTGCCCCAGTTTGCTAGG |  |
| ***cymR-*F** | ATGGCATCGTCAGCGCTTGAACTTGGCGTAGC |  |
| ***glnA*-A-R** | CTTTGATAGACGACCATTGGGATCCTTCCTCCATCAGTC |  |
| ***glnA*-B-R** | CAAACTGCTATCGACCATTGGGATCCTTCCTCCATCAGTC |  |
| ***rhO*-C-R** | GATCGGTCCGCGCAGAAAACTG |  |
| ***CS*-A-R** | CTTTGATAGACGACCATTCCCAGTTCCTTTCACCTGTCTCC |  |
| ***CS*-B-R** | CAAACTGCTATCGACCATTCCCAGTTCCTTTCACCTGTCTC |  |
| ***CS*-C-R** | GTAGGGCTTTCTGACGTCATTCCCAGTTCCTTTCACCTGTC |  |
| ***nir*-A-R** | CTTTGATAGACGACCATCCGCGTCCCAGGGTTCGT |  |
| ***nir*-B-R** | CAAACTGCTATCGACCATCCGCGTCCCAGGGTTCGT |  |
| ***nir*-C-R** | GCTTTCTGACGTCATCCGCGTCCCAGGGTTCGT |  |
| **A-*glnAsadB*-F** | GTTCGTCATGCCGATTTAATCGGCGACGGCAAGATCTTC |  |
| **A-*CSsadB*-F** | GTTCGTCATGCCGATTTAAGGCCGCCAAGCAGATAGGC |  |
| **A-*nirsadB*-F** | GTTCGTCATGCCGATTTAAGGGCGGCCTGTTCCTGTG |  |
| **B-R** | CCAAGCTCAGCTAATTAAGCTTAAACCAGAGGCGTAACGGAATCCC |  |
| **B-*rhOsadC*-F** | GAGAATCATGCCATGGCATGAAGAATTGACTCGCGCCCGG | *Nco*I |
| **B-*CSsadC*-F** | GAGAATCATGCCATGGCATGGGCCGCCAAGCAGATAGGC | *Nco*I |
| **B*-nirsadC*-F** | GAGAATCATGCCATGGCATGGGGCGGCCTGTTCCTGTG | *Nco*I |
| **C-R** | AACTAGTCTAGACTACTTAGATAATTGCTGAGCGCTCTCCG | *Xba*I |
| ***ssuE*-F** | ATGCGTGTCATCACCCTGGC |  |
| ***ssuE*-R** | CTAGTCTAGACTACTTACGCATGGGCATTACCTCGCAGAG |  |
| ***rutF*-F** | ATGAACATTGTCGATCAACAAACTTTTCGCGATGCG |  |
| ***rutF*-R** | CTAGTCTAGACTACGTTAACAAGCAGGGCGCATCAGCGC |  |
| ***cogA*-F** | ATGCGAAAGCTCACTGTTGTTACC |  |
| ***cogA*-R** | CACCAATAACTGCCTTTTAGCCGTTTCCGTCGTGTC |  |
| ***sutR*-F** | ATGAACGCACGTGTTATCAGGGTCGTGGTGGTGTCCGG |  |
| ***sutR*-R** | CGCGGATGAATGGTGATTAAATCTTTAAACAGACCACCGC |  |
| ***psuK*-F** | ATGAACGCACGTGTTATCAGGGTCGTGGTGGTGTCCGG |  |
| ***psuK*-R** | CCAATAACTGCCTTTCAGGCCGCGCGGGCGACCGGGATATGAAAG |  |
| **RT-16S-F** | CCGTATACGCCCTTTGGGGGAAAG |  |
| **RT-16S-R** | GTAGGAGTCTGGGCCGTGTCTCAG |  |
| **RT-*sadA*-F** | CTGGGAGCAACACAGTCGTC |  |
| **RT-*sadA*-R** | GCACTAGATCGTGGTTGGGG |  |
| **RT-*sadB*-F** | ACGCAGCCAGCCAAGTATG |  |
| **RT-*sadB*-R** | CTTCTCGGGATAAAGCGCGG |  |
| **RT-*sadC*-F** | CAGACAGCATCGAGATCGCC |  |
| **RT-*sadC*-R** | CCTCCAGCCGATTCACCAC |  |
| **RT-*ssuE*-F** | GCACTCAAGACCTTCACCGA |  |
| **RT-*ssuE*-R** | GTAGCACCACTTTGCCTTGC |  |
| **RT-*rutF*-F** | TGACTGCCGTATCAGCCAG |  |
| **RT-*rutF*-R** | TAACAAGCAGGGCGCATCAG |  |
| **RT-*cogA*-F** | TACTTGCGAGCAGTTGTCGT |  |
| **RT-*cogA*-R** | GGCCTTGCACACTGGAGTAA |  |
| **RT-*sutR*-F** | GTGTTCCCTTTATCCGGCCT |  |
| **RT-*sutR*-R** | CATCTGTTCGTCGTATGCCTG |  |
| **RT-*psuK*-F** | ATCACCAGTCGCTCAAGGG |  |
| **RT-*psuK*-R** | ACTCGACACTGGCGTACAG |  |
| **H-*sadC*-F** | CACCACCACCACATGACGTCAGAAAGCCCTACCC |  |
| **H-*sadC*-R,** | CCGGATCTCACTCGAGTTAGATAATTGCTGAGCGCTCTCCG |  |
| **H-*ssuE*-F** | CACCACCACCACATGCGTGTCATCACCCTGGC |  |
| **H-*ssuE*-R** | CCGGATCTCACTCGAGTTACGCATGGGCATTACCTCGCAG |  |
| **H-*rutF*-F** | CACCACCACCACATGAACATTGTCGATCAACAAACTTTTCGCG |  |
| **H-*rutF*-R** | CCGGATCTCACTCGAGTTAACAAGCAGGGCGCATCAGC |  |
| **H-*cogA*-F** | CACCACCACCACATGCGAAAGCTCACTGTTGTTACC |  |
| **H-*cogA*-R** | CCGGATCTCACTCGAGTTAGCCGTTTCCGTCGTGTC |  |
| **H-*sutR*-F** | CACCACCACCACATGAATGAAGTGATTAAATCTTTAACAGACC |  |
| **H-*sutR*-R** | CCGGATCTCACTCGAGTTATTTTTCAACTTTAAATCCTTGTTTTTCAAG |  |
| **H-*psuK*-F** | CACCACCACCACATGAACGCACGTGTTATCAGGGTCGTGGTGG |  |
| **H-*psuK*-R** | CCGGATCTCACTCGAGTTAGGCCGCGCGGGCGACCGGGATATG |  |

# Supplementary Table 3 DNA sequences of promoters, genes, and pIND4-P*_glnA_*-*sfGFP*-P_Q5_-*RFP* used in this study

| **Name** | **Sequence** (**5' to 3'**)* |
| --- | --- |
| **Promoters** |  |
| P*_glnA_* | TCGGCGACGGCAAGATCTTCATCCTGCCGGTCGAACAGGCCATCCGCATCCGCACCGGCGAGACCGGCGACGACGCGGTCTGATCCCGCCCACCCGCACCATGAACCAATGACCCATGCCCGGCGGCGGCGCCGGGCAGACTGATGGAGGAAGGATCCCA |
| P*_rhO_* | AAGAATTGACTCGCGCCCGGATTCTGCCTATGTGACCCGCAACCGCCGTCCGGTGGGGTTTTTCCTGACACAGTTTTCTGCGCGGACCGATC |
| P*_CS_* | GGCCGCCAAGCAGATAGGCGAGTCGCACGGTCTGGGGCTGGGCAAGGTGGCGGCGCCGCTTCGTGCGGCCCTCGCGGGGCGCAGTTCGACCCCTAGCGTGTTCGATATGATGTTGGCGCTTGGCCGCGACGAAACGCTGGCCCGGATGCAGGATCAAGCGGGCTGAGACCCGCGCCCATCACCCGGCCCGCGGGCCGGAGACAGGTGAAAGGAACTGGGA |
| P*_nir_* | GGGCGGCCTGTTCCTGTGCGGCGACCGCAAGCGGCCCAAGGACAAGGGCCAGGGCTGCCGAGGCCAGTAGGCCGGGTCTGGCGAATGGGGTCCTTTGTCTCATCTGGCTTTCCTATTTGCTGGCGCGCGTTCACGCCGTTCCTGGGCGCAATCCGCGCGCGGGGCTTTGACCTTTGTTAAGGCCGAGGTGCGGCATTTGCGCGATCAGCGGGACGAACCCTGGGACGCGG |
| **Genes** |  |
| *sfGFP* | ATGGTGCGTATAGGTGAAGAACTGTTCACCGGTGTTGTTCCGATCCTGGTTGAACTGGACGGTGACGTTAACGGTCACAAATTCTCTGTTCGTGGTGAAGGTGAAGGTGACGCTACCAACGGTAAACTGACCCTGAAATTCATCTGCACCACCGGTAAACTGCCGGTTCCGTGGCCGACCCTGGTTACCACCCTGACCTACGGTGTTCAGTGCTTCGCTCGTTACCCGGACCACATGAAACAGCACGACTTCTTCAAATCTGCTATGCCGGAAGGTTACGTTCAGGAACGTACCATCTCTTTCAAAGACGACGGTACCTACAAAACCCGTGCTGAAGTTAAATTCGAAGGTGACACCCTGGTTAACCGTATCGAACTGAAAGGTATCGACTTCAAAGAAGACGGTAACATCCTGGGTCACAAACTGGAATACAACTTCAACTCTCACAACGTTTACATCACCGCTGACAAACAGAAAAACGGTATCAAAGCTAACTTCAAAATCCGTCACAACGTTGAAGACGGTTCTGTTCAGCTGGCTGACCACTACCAGCAGAACACCCCGATCGGTGACGGTCCGGTTCTGCTGCCGGACAACCACTACCTGTCTACCCAGTCTGTTCTGTCTAAAGACCCGAACGAAAAACGTGACCACATGGTTCTGCTGGAATTCGTTACCGCTGCTGGTATCACCCACGGTATGGACGAACTGTACAAAGATGCATGCCAGTTCTAA |
| *RFP* | ATGGTGAGCGAGCTGATTAAGGAGAACATGCACATGAAGCTGTACATGGAGGGCACCGTGAACAACCACCACTTCAAGTGCACATCCGAGGGCGAAGGCAAGCCCTACGAGGGCACCCAGACCATGAGAATCAAGGCGGTCGAGGGCGGCCCTCTCCCCTTCGCCTTCGACATCCTGGCTACCAGCTTCATGTACGGCAGCAAAACCTTCATCAACCACACCCAGGGCATCCCCGACTTCTTTAAGCAGTCCTTCCCCGAGGGCTTCACATGGGAGAGAGTCACCACATACGAAGACGGGGGCGTGCTGACCGCTACCCAGGACACCAGCCTCCAGGACGGCTGCCTCATCTACAACGTCAAGATCAGAGGGGTGAACTTCCCATCCAACGGCCCTGTGATGCAGAAGAAAACACTCGGCTGGGAGGCCTCCACCGAGACCCTGTACCCCGCTGACGGCGGCCTGGAAGGCAGAGCCGACATGGCCCTGAAGCTCGTGGGCGGGGGCCACCTGATCTGCAACTTGAAGACCACATACAGATCCAAGAAACCCGCTAAGAACCTCAAGATGCCCGGCGTCTACTATGTGGACAGAAGACTGGAAAGAATCAAGGAGGCCGACAAAGAGACCTACGTCGAGCAGCACGAGGTGGCTGTGGCCAGATACTGCGACCTCCCTAGCAAACTGGGGCACAGATGA |
| *sadA* | ATGGTCGTCTATCAAAGGAGAGGGATCCAAATGAAATCTGTCCAATCGGCTACTGCGGCGTCAGATGGTGCGCATTCGTTCGTTCCAGAAATCTCACAGCCGAACCTTGACCTCCTCGGCCGCGCCCAATCGCTCTACTCACTTATCCATGAACATGCCCCAGACAGCGATCGTGACCGCCGTGTCTCCGAAGTGGTGATCGATGGGCTGGAAGAACTTGACCTCTTCCAGGTCTGCACTCCTCGTCGCTATGGCGGATTCCAGAGCAATTTTCGAACGCTGTTCGAGCTCACCGCCGAAATCGCACGGGGAGATGGGGGTACAGCTTGGGCGTTTGCGTTGCTCAACTCAAACGCCTGGGACGTCGGCACCTACTCACGCGAGGCCCAGGACGACATTTGGGGCGCGAATCCGCGTGCACGCATCACCACTGTAACGAATCCTCCTGCCGGCCCTACCGCATCGGCGCGCAAGGTTGACGGTGGCTATGTAATCAGCGGCCGTTGGCCGTACGCGTCAGGGTCCCTCCACGCACAATGGGCGGAGCTCGGGTTCGTCGTCGAGATTGATGGCGCACCGGTACGGATGATGACTCTCGTTCCGATGGACGAGGTCACGCTTGAAGACACGTGGTATGTCGCAGGAATGCGTGGCTCTGGGAGCAACACAGTCGTCGGCACAGAGGTTTTCGCGCCGGACTACCGCACTCAAAGCTTTGACAATCTAGTCGAAGGAAACTACGCGTCCGAGTTCACCGATGAGCTCGAATATCTCACGCCTATGGTCCCCAACCACGATCTAGTGCTCGTTGGGGCTCAAGTCGGACTTGCTCAGGCAGCTCTGGATTATGCTCTCGAAAAGCTTCCGACGCGCGGCGTCACAAACACAAAGTATGCAAAGGGGAGCGACGCGCCGACGAACCAGATTGCGGTCGCCGAGGCTGCGAACGCGATTGACACTGCACGCATGCTTGGGCGGCGAGCTAGTTATGACATCGATGCCGCCGCGGTAACCAATCGTGGTCAAATCGATTGGGCAACGCGCGCACGTATTCGGATGGATGCGGCGACCATAGCGGTGCTATGTCGAGAGTCCATCGATAAAATGCTCACTGCTATCGGTTCGGCCGCCTTTGCCTCGGTGAACCCACTTCAGCAGATATGGAGGGACTCGGAAACGGCAAGCCGTCATGCGTTGGTGAACGTGGGCGTTTCGAAGGAAACGTACGGGAAGTCGCTGCTCGGCATCGACGAGTTCGTCATGCCGATTTAA |
| *sadB* | ATGGTCGATAGCAGTTTGCCCCCTGACATCTCCGCCCTCCTGGAGAGGATCGATGAGATCCGCCCGCTGATTGAGAAGAACGCAGCGCAAGGCGAAGCAGAACGACGCGTGTCGCAGGAGTCGATCGATGCCCTCGAGGCAATCGGAGCCTTCCGGGTGACGCAGCCAGCCAAGTATGGAGGATATGAAGGTGATTCTCGTGCGCAGGTCGACGTGGGCGCAGCCGTCGGCAAGGGCGACGGCGGGACTGCCTGGGTCGTCGCGCTCACCAACATCGCGAACTGGCTGACCGCGCTTTATCCCGAGAAGGCGCAGGATGATGTGTGGGGTGAGGATCGCAACGCAAAGGTCTCCGTGGTCCTAGCCACAAACGGTAAGACCCAGCGCGTTGATGGTGGGTACCTCGTCAGCGGGGAGTGGAGCTACAACTCGGCATCGTGGCACACGCAGTGGGCGATTCTCGGGGCCGAGCTCGTCGACGAGAATGGTGACTTCGTTGACACCGCGCAACTGCTGATCCCGCGCTCGGATCTCGGCTTCAAGGACATCTGGCATGTGGCCGGCATGCGTTCGAGCGGAAGCAACGCGCTCAGCGCGACTGACGTTTTCGTTCCCGATCACCGTGTGATGCGTGGGGAACCTGCCTTGCGAGGCACCTACCCTGGCACGACCGAGGACACTCCCGCGGTCTACCGGGCGGGGTGGATTCCCGTGCTGAACATCATTCTCGTCGGCCCGCAGCTCGGCATGGGCCGTGCCGTGCTAGAGAGGGTGATCTCGAAGGCAGACTCGAAAGCCATCGCATACACAAGCTTCGAGCGACAGAGTGACTCGATCGCATTCCAACTCGACATCGCCAAGGCCGCGCTGCTGCTGGAAGCTGCGGAAGGGTTCGCCCACCGCGCCACTGACGAGATCGACATCCCCGCTGCTCAGGGCGTCTATCCGGACTACTTGACCCGAGCTCGAAATCGTGCGTACGTGGGCTGGATCGTCGAGCACACCGCTCGCGCCATTGAGATGTTGCTCACCGCGCACGGGTCGGGAGCTTTCGCGGAAGTGAACCCGTTGCAGCGCCTTTGGCGTGACCAGGCAGTCGCGTCACGGCACGCGTTCGTTCTTCCGGCGCTCGGTTACGAGCTTTACGGGAAGGCCCTTCTCGGCCGCGAAGATGGGGATTCCGTTACGCCTCTGGTTTAA |
| *sadC* | ATGACGTCAGAAAGCCCTACCCCGAAGCACGCGCAGGAGTCGAGCAGTGGGGGTGGCGCTCTGGTCCTGGACACGCTCTCCGCAGACGATTTCCGGGCGATCTTCCGTCGGCATCCTGCTGGCGTGACCGTCATAACGGCAGACGCAGGAACCGGCCCAGTCGCCCTCACGGCCACATCGGTCGCCTCTATGAGCGCCGATCCGCCGCTGCTAATCTTCTCCGTGTCATCGTCGTCCTCGAGTGCGCGCATCCTGAAAGATGCCAATACTGTCGTCGTCCACTTCATCGGCCCAGACAGCATCGAGATCGCCAAACTTGGCGCGACGAGCGGAATCGACCGATTCGCGGACTCGACCATCTGGTCCCGCCTCCCAACCGGGGAAGTGGTGTTTGATGCGGTTCGCACGTGGATCCGCGCGCGGGTGGTGAATCGGCTGGAGGCGAGCGGATCGACCATCGTGATCGCACAGGCGATCGAGTCGAACTACACGGCGGCAGAACTCGATCAGCAACCGGAGCGCGAAGGACTCGTCTTCATGAACCGAAGCTGGCACCAGGTCGGAGAGCGCTCAGCAATTATCTAA |
| *ssuE* | ATGACGTCAGAAAGCCCTACCCCGAAGCACGCGCAGGAGTCGAGCAGTGGGGGTGGCGCTCTGGTCCTGGACACGCTCTCCGCAGACGATTTCCGGGCGATCTTCCGTCGGCATCCTGCTGGCGTGACCGTCATAACGGCAGACGCAGGAACCGGCCCAGTCGCCCTCACGGCCACATCGGTCGCCTCTATGAGCGCCGATCCGCCGCTGCTAATCTTCTCCGTGTCATCGTCGTCCTCGAGTGCGCGCATCCTGAAAGATGCCAATACTGTCGTCGTCCACTTCATCGGCCCAGACAGCATCGAGATCGCCAAACTTGGCGCGACGAGCGGAATCGACCGATTCGCGGACTCGACCATCTGGTCCCGCCTCCCAACCGGGGAAGTGGTGTTTGATGCGGTTCGCACGTGGATCCGCGCGCGGGTGGTGAATCGGCTGGAGGCGAGCGGATCGACCATCGTGATCGCACAGGCGATCGAGTCGAACTACACGGCGGCAGAACTCGATCAGCAACCGGAGCGCGAAGGACTCGTCTTCATGAACCGAAGCTGGCACCAGGTCGGAGAGCGCTCAGCAATTATCTAA |
| *rutF* | ATGAACATTGTCGATCAACAAACTTTTCGCGATGCGATGTCCTGCATGGGCGCGGCGGTCAATATTATCACCACGGACGGTCCAGCCGGGCGCGCCGGGTTCACCGCCAGCGCCGTCTGCAGTGTGACCGATACGCCGCCCACATTACTGGTGTGCCTGAATCGTGGGGCGTCCGTCTGGCCGGCATTCAATGAAAATCGAACGCTGTGTGTAAATACACTTAGCGCCGGGCAGGAGCCGCTTTCAAACCTTTTTGGCGGCAAAACGCCCATGGAACACCGCTTTGCCGCCGCCCGCTGGCAGACGGGGGTGACCGGATGCCCGCAACTGGAAGAGGCTCTGGTTTCGTTTGACTGCCGTATCAGCCAGGTGGTGAGCGTCGGCACCCACGACATTCTGTTTTGCGCCATCGAAGCGATTCATCGTCACACCACACCCTACGGGCTGGTGTGGTTCGATCGCAGTTATCACGCGCTGATGCGCCCTGCTTGTTAA |
| *cogA* | ATGCGAAAGCTCACTGTTGTTACCGCAGGCCTTTCTAACCCGTCCACCACTCGCTCCGTGGCGGATCAACTCACCAAGGCAGTCCAAACCGCTGTTTCTGCTCGTGGCGAATCCCTGGATATTGAAGTAATTGAAATCCGAGACCTGATCTTTGATCTCGCTACCTCATTCACAAGCGCCGGCATGAGCTCCCCAGCACTTGACGCTGCAAAACAGCGCCTTGCTGAATCCGATGGCCTGATCGCTGTTACCCCAGTATTTACCGCGAGCTACTCCGGCATCTTCAAGATGTTCTTTGATGTCCTGGACCCCAAGACCATTGTGGGTCTGCCCACCATCATTGCGGCATCTGCTGGAACGGCACGCCACTCATTGGTTCTCGACCACGCCATCCGACCACTGTTTACCTACTTGCGAGCAGTTGTCGTACCCACCGGCGTGTTCGCAGCCACGGAAGATTTCGGCACTGAAGCTGGCGCAGACATTGAACGTCGCGTGAACCGCGCAGCTGGCGAATTAGCGACACTCATGTTGCAGGATTACTCCAGTGTGCAAGGCCTTGGGGGCGCAACCGCGAACCAAGACGCTGACCTTTCCTTCCGTCGCACCACTGGCGTGACCCCGGGAGAGAACTTCAGCAGCTTTGCCGATCTTCTCAAAGGACACGACGGAAACGGCTAA |
| *sutR* | ATGAATGAAGTGATTAAATCTTTAACAGACCACCGCTCGATTCGCAGCTACACAGATGAGCCTGTAGCTCAGGAGCAATTGGACCAAATCATTGAAGCGGTGCAATCAGCCCCTTCTTCTATCAACGGACAGCAAGTGACTGTGATCACAGTCCAGGATAAAGAGCGCAAAAAGAAAATCTCCGAGCTGGCAGGCGGCCAGCCATGGATCGATCAAGCTCCTGTTTTCCTGCTGTTCTGCGCAGATTTCAATCGGGCCAAAATTGCGCTTGAAGATCTGCATGATTTCAAAATGGAAATCACAAATGGATTGGAATCTGTTCTTGTCGGCGCAGTAGACGCTGGTATTGCCCTCGGCACAGCAACAGCAGCGGCTGAGTCACTCGGACTTGGCACAGTTCCGATCGGTGCGGTTCGCGGAAACCCTCAAGAGCTGATCGAACTGCTTGAGCTTCCAAAATACGTGTTCCCTTTATCCGGCCTTGTCATTGGGCATCCTGCCGACCGTTCAGCGAAAAAACCGCGCTTGCCGCAGGAAGCTGTCAATCATCAGGAAACTTATTTGAATCAGGATGAGCTGACGTCTCACATTCAGGCATACGACGAACAGATGTCTGAATACATGAATAAACGGACAAACGGAAAAGAAACAAGAAACTGGTCACAGAGCATCGCTTCCTACTATGAGCGCCTGTACTATCCGCACATCCGTGAAATGCTTGAAAAACAAGGATTTAAAGTTGAAAAATAA |
| *psuK* | ATGAACGCACGTGTTATCAGGGTCGTGGTGGTGTCCGGCAGCCTGCGGGCCCCATCACGCACCCATGGTCTGCTGCAGGCCCTGGTGGAGCGGCTGCCGGCCGTGTTGCCGAAGCTGGAGGTGCACTGGGTGCGCATCGCCGAACTGTCCGCCAGCCTGGCCGGTTCGCTGGAGCGCGATAGCGCCTCGGCTGACCTGCAACCGCATTTGCAGGCCATCGAGCAAGCCGACCTGCTACTGGTCGGCAGCCCGGTGTATCGCGCGTCCTACACCGGTTTGTTCAAGCACCTGTTCGACCTGGTCGATCACCAGTCGCTCAAGGGCGTGCCCGTGGTGCTTGCCGCCACCGGTGGTAGCGAGCGACATGCCTTGATGATCGATCACCAGTTGCGCCCGCTATTCGCTTTCTTCCAGGCCCACACCCTGCCGTACGGCCTGTACGCCAGTGTCGAGTCTTTCGACGATCAGCGCCTGGCTGACCCTGCGCAGTTCGAACGGATCGAGCGGGTACTCGATACCGTCGGCGCCTTCTTTCATATCCCGGTCGCCCGCGCGGCCTGA |
| **plasmid** |  |
| pIND_4_-P*_glnA_*-  *sfGFP*-P_Q5_-  *RFP* | GGCCGCGCGAATTCGAGCTCGGTACCGACGTAGCCCAGCGCGTCGGCCAGCTTGCAATTCGCGCTAACTTACATTAATTGCGTTGCGCTCGGCGACGGCAAGATCTTCATCCTGCCGGTCGAACAGGCCATCCGCATCCGCACCGGCGAGACCGGCGACGACGCGGTCTGATCCCGCCCACCCGCACCATGAACCAATGACCCATGCCCGGCGGCGGCGCCGGGCAGACTGATGGAGGAAGGATCCCAATGGTGCGTATAGGTGAAGAACTGTTCACCGGTGTTGTTCCGATCCTGGTTGAACTGGACGGTGACGTTAACGGTCACAAATTCTCTGTTCGTGGTGAAGGTGAAGGTGACGCTACCAACGGTAAACTGACCCTGAAATTCATCTGCACCACCGGTAAACTGCCGGTTCCGTGGCCGACCCTGGTTACCACCCTGACCTACGGTGTTCAGTGCTTCGCTCGTTACCCGGACCACATGAAACAGCACGACTTCTTCAAATCTGCTATGCCGGAAGGTTACGTTCAGGAACGTACCATCTCTTTCAAAGACGACGGTACCTACAAAACCCGTGCTGAAGTTAAATTCGAAGGTGACACCCTGGTTAACCGTATCGAACTGAAAGGTATCGACTTCAAAGAAGACGGTAACATCCTGGGTCACAAACTGGAATACAACTTCAACTCTCACAACGTTTACATCACCGCTGACAAACAGAAAAACGGTATCAAAGCTAACTTCAAAATCCGTCACAACGTTGAAGACGGTTCTGTTCAGCTGGCTGACCACTACCAGCAGAACACCCCGATCGGTGACGGTCCGGTTCTGCTGCCGGACAACCACTACCTGTCTACCCAGTCTGTTCTGTCTAAAGACCCGAACGAAAAACGTGACCACATGGTTCTGCTGGAATTCGTTACCGCTGCTGGTATCACCCACGGTATGGACGAACTGTACAAAGATGCATGCCAGTTCTAAGCTTAATTAGCTGAGCTTGGACTCCTGTTGATAGATCCAGTAATGACCTCAGAACTCCATCTGGATTTGTTCAGAACGCTCGGTTGCCGCCGGGCGTTTTTTATTGGTGAGAATCCAAGCTAGCTTGGCGAGATTTTCAGGAGCTAAGGAAGCTAAAATGGAGAAAAAAATCACTGGATATACCACCGTTGATATATCCCAATGGCATCGTCAGCGCTTGAACTTGGCGTAGCGCTCGCGGGCGATTTCCAGGGTCGAGTTGCGGACGCGCTCGAAGCGCTCCTTGTCCTTCTGCCACAGCGAGCGGACCGCCAGGCCGCGGACCGAGTTGAAGATCAGCCACAGGATGTCCTCCGCGTCGTCGCGGCTCAGGCCGCGCGACACCAGCACGCCCAGCCACATATCCTCGACGACGAAGCGGTTGCGCTCGACGGTGCGCTGGATGCCCTCGCGCAGCGCCGGGTCGCGGTCGGCGGCCACGATCAGGTCCAGCGAGATCGAGAAGTCGTCGTCCAGGAAGAACTCGGCGGCGTCGTCCAGCATCTGCTGGATGACGTCGTCCTCGGGCTTCAGCTTGGCCAGGCGGGCGCGGCTGCGCTCGGTGATCTGCTCGTACAGCCACTCGAAGGTGGCCAGCAGCAGTTCCAGCTTGGTCGGGAAGTGGTGCGACTGGGCGCCGCGGCTGACGCCGGCGGCGCCCGGGACGTCGGCGATGCGGAAGCCGGCATAGCCCTTCTCGCGCAGGACGCCCAGGGCGGCGGCGATCAGCTTGCCCTGGGTTTCCATCGCGCGCTCGGCCTGGGTGCGGCGCTTCGGCGACATGATGACCATTCGGGGTCTCTCCCTGCTAGCATCAGGGTTATTGTATCATGAGCGGATACATATGTCAATGTACCGGAACAAACAGACAATCTGGTCTGTTTGTACATGGTTGACGGATCGCCGCGAAGCCGCTAACTGCGCCGGTTAACAAACAGACAATCTGGTCTGTTTGTAACTAGTAGACAGGAGAAGGCATGCCATGGCATGTATGGTGAGCGAGCTGATTAAGGAGAACATGCACATGAAGCTGTACATGGAGGGCACCGTGAACAACCACCACTTCAAGTGCACATCCGAGGGCGAAGGCAAGCCCTACGAGGGCACCCAGACCATGAGAATCAAGGCGGTCGAGGGCGGCCCTCTCCCCTTCGCCTTCGACATCCTGGCTACCAGCTTCATGTACGGCAGCAAAACCTTCATCAACCACACCCAGGGCATCCCCGACTTCTTTAAGCAGTCCTTCCCCGAGGGCTTCACATGGGAGAGAGTCACCACATACGAAGACGGGGGCGTGCTGACCGCTACCCAGGACACCAGCCTCCAGGACGGCTGCCTCATCTACAACGTCAAGATCAGAGGGGTGAACTTCCCATCCAACGGCCCTGTGATGCAGAAGAAAACACTCGGCTGGGAGGCCTCCACCGAGACCCTGTACCCCGCTGACGGCGGCCTGGAAGGCAGAGCCGACATGGCCCTGAAGCTCGTGGGCGGGGGCCACCTGATCTGCAACTTGAAGACCACATACAGATCCAAGAAACCCGCTAAGAACCTCAAGATGCCCGGCGTCTACTATGTGGACAGAAGACTGGAAAGAATCAAGGAGGCCGACAAAGAGACCTACGTCGAGCAGCACGAGGTGGCTGTGGCCAGATACTGCGACCTCCCTAGCAAACTGGGGCACAGATGAGTGATGGCTTCCATGTCGGCAGAATGCTTAATGAATTACAACAGTACTGCGATGAGTGGCAGGGCGGGGCGTAATTTTTTTAAGGCAGTTATTGGTGCCCTTAAACGCCTGGGGTAATGACTCTCTAGCTTGAGGCATCAAATAAAACGAAAGGCTCAGTCGAAAGACTGGGCCTTTCGTTTTATCTGTTGTTTGTCGGTGAACGCTCTCCTGAGTAGGACAAATCCGCCGCTAGGAGCTTGCGGCCCTGGCGTAATAGCGAAGAGGCCCGCACCGATCGCCCTTCCCAACAGTTGCGCAGCCTGAATGGCGAATGCGATTTATTCAACAAAGCCGCCGTCCCGTCAAGTCAGCGTAATGCTCTGCCAGTGTTACAACCAATTAACCAATTCTGATTAGAAAAACTCATCGAGCATCAAATGAAACTGCAATTTATTCATATCAGGATTATCAATACCATATTTTTGAAAAAGCCGTTTCTGTAATGAAGGAGAAAACTCACCGAGGCAGTTCCATAGGATGGCAAGATCCTGGTATCGGTCTGCGATTCCGACTCGTCCAACATCAATACAACCTATTAATTTCCCCTCGTCAAAAATAAGGTTATCAAGTGAGAAATCACCATGAGTGACGACTGAATCCGGTGAGAATGGCAACAGCTTATGCATTTCTTTCCAGACTTGTTCAACAGGCCAGCCATTACGCTCGTCATCAAAATCACTCGCATCAACCAAACCGTTATTCATTCGTGATTGCGCCTGAGCGAGACGAAATACGCGATCGCTGTTAAAAGGACAATTACAAACAGGAATCGAATGCAACCGGCGCAGGAACACTGCCAGCGCATCAACAATATTTTCACCTGAATCAGGATATTCTTCTAATACCTGGAATGCTGTTTTCCCGGGGATCGCAGTGGTGAGTAACCATGCATCATCAGGAGTACGGATAAAATGCTTGATGGTCGGAAGAGGCATAAATTCCGTCAGCCAGTTTAGTCTGACCATCTCATCTGTAACATCATTGGCAACGCTACCTTTGCCATGTTTCAGAAACAACTCTGGCGCATCGGGCTTCCCATACAATCGATAGATTGTCGCACCTGATTGCCCGACATTATCGCGAGCCCATTTATACCCATATAAATCAGCATCCATGTTGGAATTTAATCGCGGCTTCGAGCAAGACGTTTCCCGTTGAATATGGCTCATAACACCCCTTGTATTACTGTTTATGTAAGCAGACAGTTTTATTGTTCATGATGATATATTTTTATCTTGTGCAATGTAACATCAGAGATTTTGAGACACAACGTGGCTTTGTTGAATAAATCGAACTTTTGCTGAGTTGAAGGATCAGATCACGCATCTTCCCGACAACGCAGACCGTTCCGTGGCAAAGCAAAAGTTCAAAATCACCAACTGGTCCACCTACAACAAAGCTCTCATCAACCGTGGCTCCCTCGAGGCTCCGCGCCCTCGATCCGACCGCCCCCCACAAGGCTGGGGCGGATCGGGGGCGCACGGCCTGAAGGGTGCAGGGGAACCCTGCCGCACGCAAACTCGCAGAGTTTGCATAGTGTGCGCCATTCAGATACCTTCATGGCAGGCCCCACCGTGGATTTTGCTGTCATGGTTCGATTCATCACGACGCCCGCAACGCCGACTGCACAGCGGCCCGCACCACGAACGCCTGCGCAGCAGGAAGCTGCGGCTGAACTGAAGGCTGCGCGAACTGCCGAAGCAGCCCGCCGCCTCGACACCCGGCGCAAGGTGGTGCTCGGGGGCGCGCTCCTGGCACTGGCAGAACGTGATGCAGCAGCAGCCGCGATGCTGGCCCGGCTGAAGCAGATGCTGACCCGCCCAGCAGATCGGAAGCTGTTCGCGGATCTACCCGATGGCTGACGGTGCAATCTTCCACCTCGATGTGCGCACGGTCAGCCGGTCAGATGGCCGCTCGGCTGTCGCCGCTGCCGCCTATCGGTCGGCCAGCCGTCTGCACGATGCACGCACCGGCCTGACCCACGACTTCGCGCGCAAGCGGGGCGTCTTGACAACCTTCATCGCCGCCCCGGACGGCTGCGACTGGATCACAGACAGGGACACGCTCTGGAACGCGGCTGAGGCGGCAGAGAAGCGCAAGAACAGCACCGTGGCCCGTGAGTGGCTGGTGGCCCTCCCTGACGCTCTGGATGCTACACAGCGGGCCGACCTGGCGCGGGCGCTGGCGGTCGAACTGGCGCAGCGGTTCGGGGTGGCCGTGGATGTGGCAATCCACGCAGCTTCGGGCGAGGGCGACCAGCGGAACCATCACGCGCATTTGCTGACCACGACGCGCACCGCAGGCCCGGACGGGCTGGGCGACAAGACCCGCGTTCTGGATGCAGCCAAAACGGGGGGCGCAGAAATCTACGCCATGCGGGCTTGGTGGGCGGGCACGGTCAACGATGCACTCGCGGCCGCACAAAGCTCGGCCCGCGTCGATCACCGCCGCATAGCCGTGATCGCCGCCGATGCGCGGGCCGAGGCCGAGGCGCTGGAGAAACAGGCCAAAGCCGTCGAAACCCTGAACGCCAAGCCCGCCGACGCCGGGGGGCTGTGGAAGGGCCTTGGCAGCGCCGCCCGCGCAATATGGGAAGGCGGCATGACCGCCCTCAAATCGACCGCCGAGACAGCCAACGAGCTGCGGGAGAAGGCCAACGAGCTGCGACAGAAGGCAGCCCGCTACACCCGCGCCACCGAACCTCACCACGGCCCCGTCCTGACGGCCTTCTTGCGGCGCATGGCTCCCGTCTGGGCGGAGCAGGCCAAGGCCGAGGAAGCCCGGAGAGCCGCGCAGGAGGCCGCACAGCGCGCACTGATGGCCGAGAGGGCTGAACAGGCCCGGAAGGCCAAGGAAGCCTCTGAGGCCGCTCGCCGGGCCGCTACGGCAGCGCGAGAGGCCGAGGAAGCCCGCGAACGCTGCGCCCGCGAGGTGTTGCCGCTCATCCGCCGCGCCCGGCACGACCCGATCACCGCCGAAGTGATCAAGCGGGAGGGCATCGACCTCGACCGCCCCGACCAACAGGTGGCCCGCGACCCGATCTGGCTCATGGCCGGACGCCAAGATCAGGCGCACCCGGTCTGGCTGATCGTCCACGCCTACACCGCCGCGCGCGACAGGCACGCGAACCTGCTGGCGAGAATCGAAGCCAAAACCGCAGCCCAGATCGCCGCTGCCGCCAAACCAAAGCCCAAGCCTGCCAGTCCCGCCCCGACCGAACCCGAACCCGATCCAGACCCACCGCCCCGGCGGGATTGGGGCGGGCCGTCTGGACCCTGATCCACCCCCCATTCTCAGCAACTCCGAGCCAGCCAGAGGCGCTGGCTCGGGGCATCTGGACCCCCTTCCTCACCCCAAATTTTCACGAAAAAGGGGCGCAGCCCTTCTTGTTCAATAAGTTCTATAAGTTCAGGCGAAAATCGCCTTTCATTTTCAATGGCTTGCGCGCCTATCGGGGAGAAATGGGGCCGCAAAAGGGAGAAATGGGGCCGCAAAAGGGAGAAATGGGGCCGATAGCGGCTGTTGACAGGGGGATATTTTGCCCCCTACATTTCTCCCCATGACAAAGACCACGCAAATCGCCGCTGATCGAGCCTATGACGAGGCCAAAACCGTGCTGCCCGCAGAGGTGGCGCGGGGGGTCTACATGCGCAACGCCCCCAGCCTCGCCGCCCTCAAGCTGATGCACCTGCTGATCGGTAAGGCGGGCGGGCGTATGGCCGAACCCATTCGGCACGAAATCCGCCTCTCAGACATCCGCAAGATCGACGGGATGAAGAACCATGACAGGGCCAGCCTCACCCCCCTTTTCGCGGAATTGTCGGCGGCTGTCCTGACCCATGATGACCCCGAAAAAATGGTCGTGACCATTGGCGGCTTGCTGGACTTCGCCCGCATCGACTACCGCCACGAAGTCAGCAACGATCTGCTGGTGTCGTGGTATTTTGGCGGGGCCTTCCGCGAGATGGCCGAGAAGTCCTGCCACTGGGCGATCCTCGACCGGCAGACCGTTTTTGCCCTGTCCAGCAAGTATTCGATCCTGCTGTTCCAGCACATCGCCAGCCTCGTGAACCTCGATCACGTCGCCAGCAAGACCTTCACCGTGGCCGAACTGCGGGCCGTGCTAGGGGTGCCGCACGGAAAGATGGAGCGGTGGAACGACGTGAACCGCTTTGCGCTGACGCCCGCGATTGCCGAGATCAACCAGACCTCGCGCCTGATCCTGACCGCGACCCCGAACAAGATCGGGCGGACGGTCGCCAGCGTGACAATCACGTGGGCGGAGAAGCCTCCCGAGGGCAAGCAGGAAGCCAAGCGCGAACTGGACCGCCCCAAGGTGGGCCGAGAGGCGCGGCGCAACGGCTCAGCCGAGACCGTGGCGCGGGCCTTCCCGAGCTCGGGCAGCATCGAGTTCGATGACCACTGGAAGGCCCTCAAGCGGGCGGCGGGCTGCAACATGGACAACACCATGATTGCCGAGAAGTTCCGGGCGTGGTGCGCCAGCAAGGGGCTTTCCCTCGACGGCCGAAACATCGAGCAGGCGTTCAGCAACTTCTGCGCCACAGTGGGCAAGGTCTGAGGCCGCGCCCGCTGCCAGTGACCCGCCGCCGCCGACCGTGAGCGGGCCGCAGCCCTTCCAGCGCCGCCCAAGAGGGCGGCAAGCCCCGGTCTGCCCCCTCGTCGCCCGCAAAGCGGGCAGGGGGCAGACCGGGGCGCTGGAACCTTTGGGCTGCTTTGACGGCCAGACAGCCCCCGCCGCGCAAGCGCGCCATTCCCCCACCCGTGGCCGGGCGGGGGAATGGTAAAGCATGTGGGGGCAGTCCAGCCGTCAATCCGCGCTCCGCACCACGGCGCCTCGAGCAACACCTTCTTCACGAGGCAGACCTCTCGACGGAGTTCCACTGAGCGTCAGACCCCGTAGAAAAGATCAAAGGATCTTCTTGAGATCCTTTTTTTCTGCGCGTAATCTGCTGCTTGCAAACAAAAAAACCACCGCTACCAGCGGTGGTTTGTTTGCCGGATCAAGAGCTACCAACTCTTTTTCCGAAGGTAACTGGCTTCAGCAGAGCGCAGATACCAAATACTGTTCTTCTAGTGTAGCCGTAGTTAGGCCACCACTTCAAGAACTCTGTAGCACCGCCTACATACCTCGCTCTGCTAATCCTGTTACCAGTGGCTGCTGCCAGTGGCGATAAGTCGTGTCTTACCGGGTTGGACTCAAGACGATAGTTACCGGATAAGGCGCAGCGGTCGGGCTGAACGGGGGGTTCGTGCACACAGCCCAGCTTGGAGCGAACGACCTACACCGAACTGAGATACCTACAGCGTGAGCTATGAGAAAGCGCCACGCTTCCCGAAGGGAGAAAGGCGGACAGGTATCCGGTAAGCGGCAGGGTCGGAACAGGAGAGCGCACGAGGGAGCTTCCAGGGGGAAACGCCTGGTATCTTTATAGTCCTGTCGGGTTTCGCCACCTCTGACTTGAGCGTCGATTTTTGTGATGCTCGTCAGGGGGGCGGAGCCTATGGAAAAACGCCAGCAACGCGGCCTTTTTACGGTTCCTGGCCTTTTGCTGGCCTTTTGCTCACATGTTCTTTCCTGCGTTATCCCCTGATTCTGTGGATAACCGTATTACCGCCTTTGAGTGAGCTGATACCGCTCGCCGCAGCCGAACGACCGAGCGCAGCGAGTCAGTGAGCGAGGAAGCGGAAGAAGCTCGCACATTCAGCAGCGTTTTTCAGCGCGTTTTCGATCAACGTTTCAATGTTGGTATCAACACCAGGTTTAACTTTGAACTTATCGGCACTGACGGTTACTGATTTTGAACTTTTGCTTTGCCACGGAACGGTCTGCGTTGTCGGGAAGATGCGTGATCTGATCCTTCAACTCAGCAAAAGTTCGCCAATACGCAAACCGCCTCTCCCCGCGCGTTGGCCGATTCATTAATGCAG |

The differences between pIND_4_-P*_glnA_*-*sadA* and other plasmids were promoters and expressing genes.


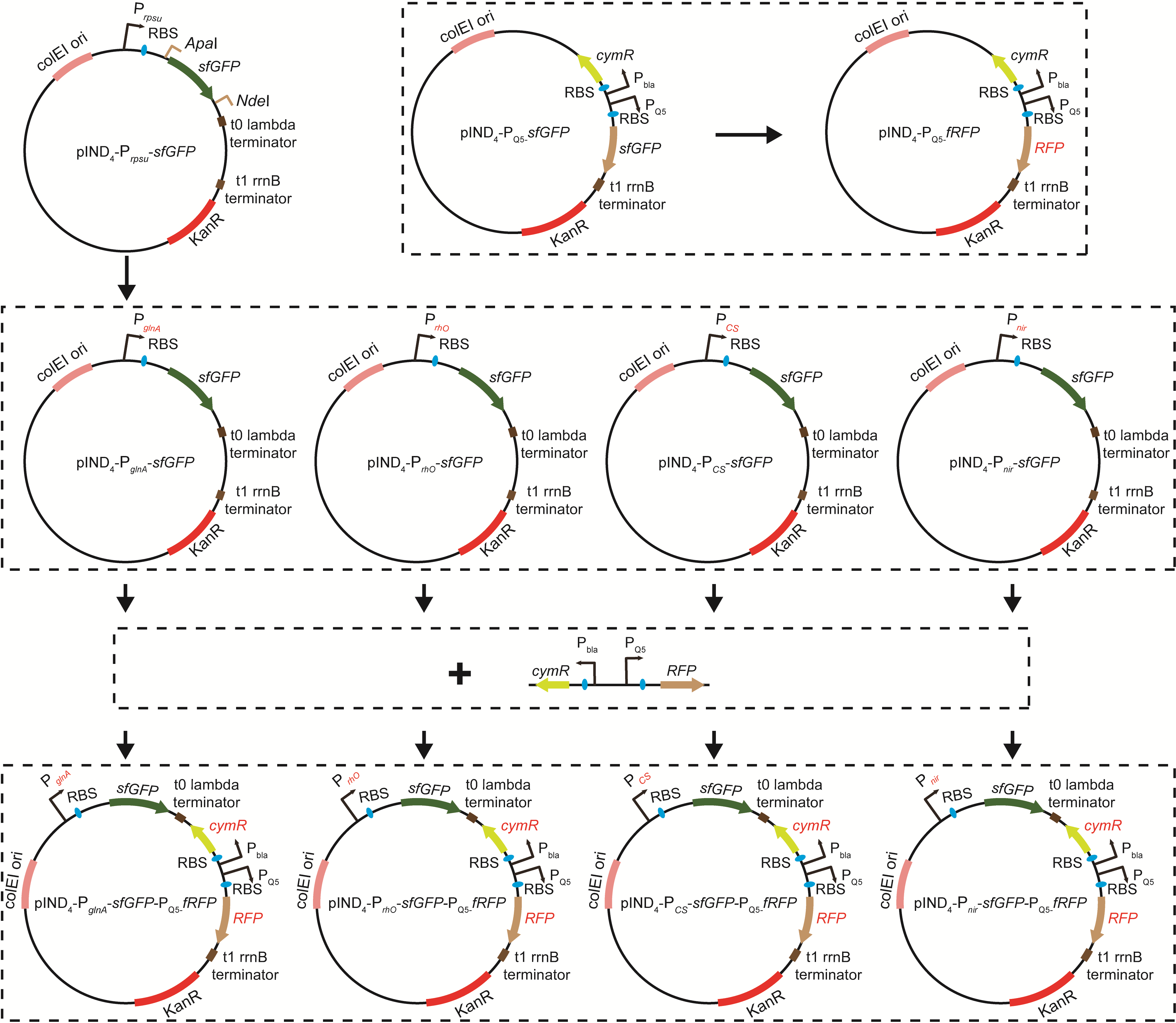


# Supplementary Figure 1. The plasmid schematic diagrams and construction processes of plasmids that used to measure strength of promoters.

The plasmids were used to generate data in Figure 1B. pIND_4_-P*_rpsu_*-*sfGFP* was the modified backbone plasmid that carried a P*_rpsu_* promoter controlled *sfGFP*. pIND_4_-P*_glnA_*-*sfGFP*-P_Q5_-*RFP*, pIND_4_-P*_rhO_*-*sfGFP*-P_Q5_-*RFP*, pIND_4_-P*_CS_*-*sfGFP*-P_Q5_-*RFP*, and pIND_4_-P*_nir_*-*sfGFP*-P_Q5_-*RFP* were the reported plasmids for P*_glnA_*, P*_rhO_*, P*_CS_* and P*_nir_* promoters, respectively, used to generate reported data in Figure 1B.


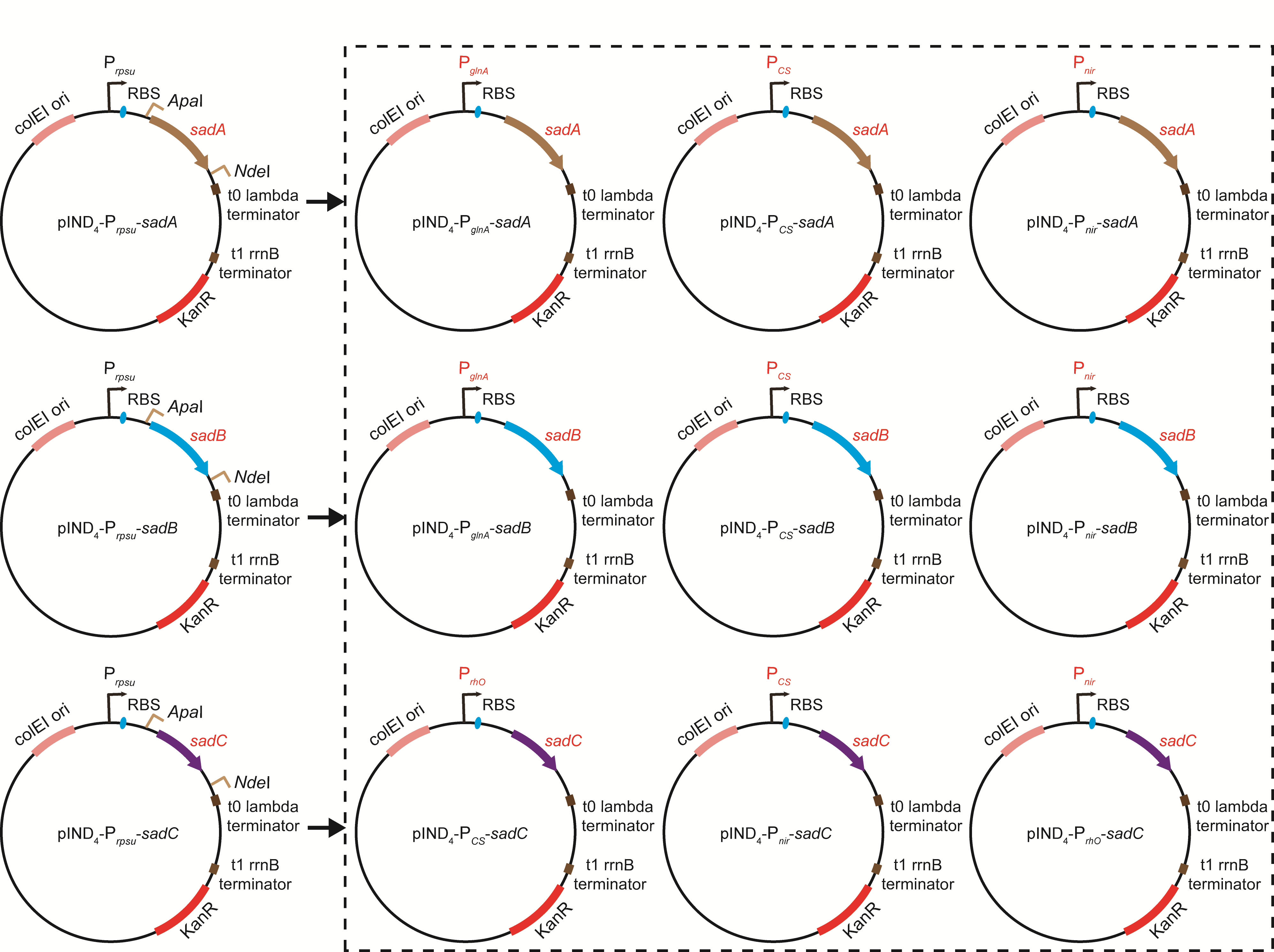


# Supplementary Figure 2. The plasmid schematic diagrams and construction processes of plasmids that used to measure strength of promoters.

The plasmids of pIND_4_-P*_glnA_*-*sadA*, pIND_4_-P*_glnA_*-*sadB*, pIND_4_-P*_rhO_*-*sadC*, pIND_4_-P*_CS_*-*sadA*, pIND_4_-P*_CS_*-*sadB*, pIND_4_-P*_CS_*-*sadC*, pIND_4_-P*_nir_*-*sadA*, pIND_4_-P*_nir_*-*sadB* and pIND_4_-P*_nir_*-*sadC* were used to generate data in Figure 1 (C-J). In addition, pIND_4_-P*_glnA_*-*sadA*, pIND_4_-P*_CS_*-*sadA*, pIND_4_-P*_nir_*-*sadA*, were used as the basis for subsequent plasmids construction.


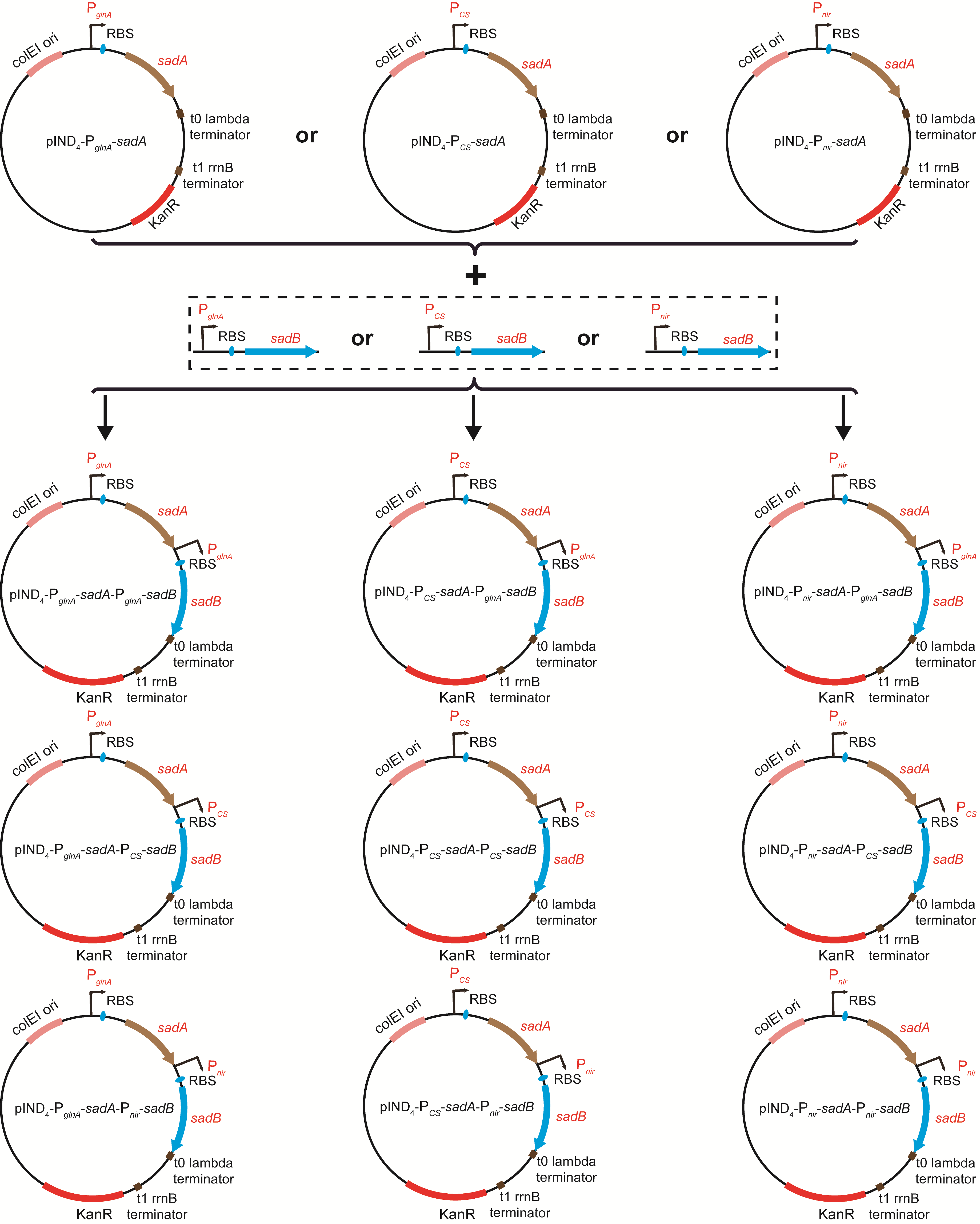


# Supplementary Figure 3. The plasmid schematic diagrams and construction processes of *sadA* and *sadB* combinatorial plasmids under the controlling of different promoters.

The nine plasmids carrying *sadA* and *sadB* genes were used to generate sulfamethoxazole degradation data in Figure 2. And pIND_4_-P*_glnA_*-*sadA*-P*_glnA_*-*sadB* was used as the basis for subsequent plasmids construction.


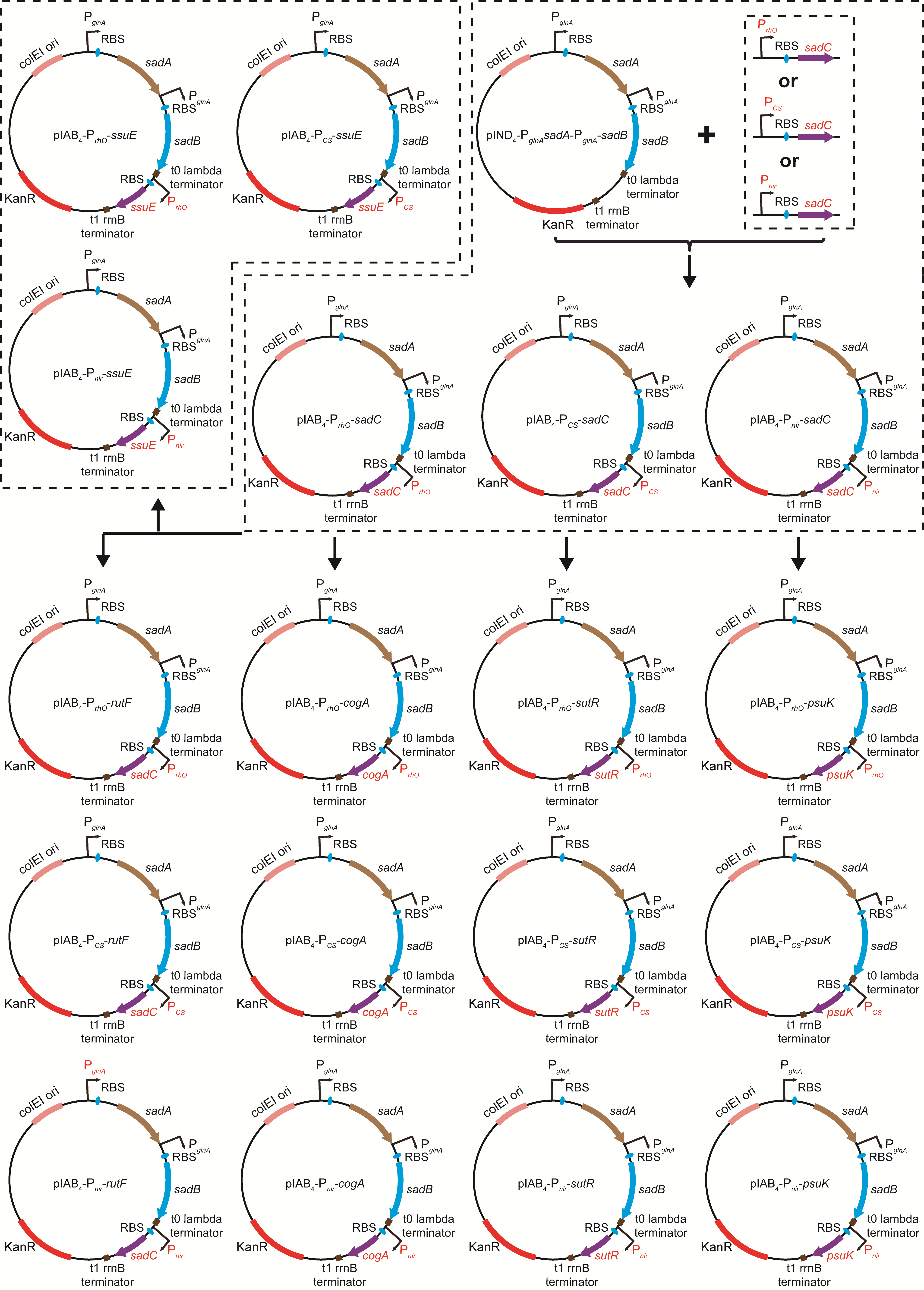


# Supplementary Figure 4. The plasmid schematic diagrams and construction processes of plasmids harboring gene *sadA*-sadB-*fmnR*.

The eighteen plasmids carrying *sadA*, *sadB* and *fmnR* genes were used to generate sulfamethoxazole degradation data in Figure 3(A-C).


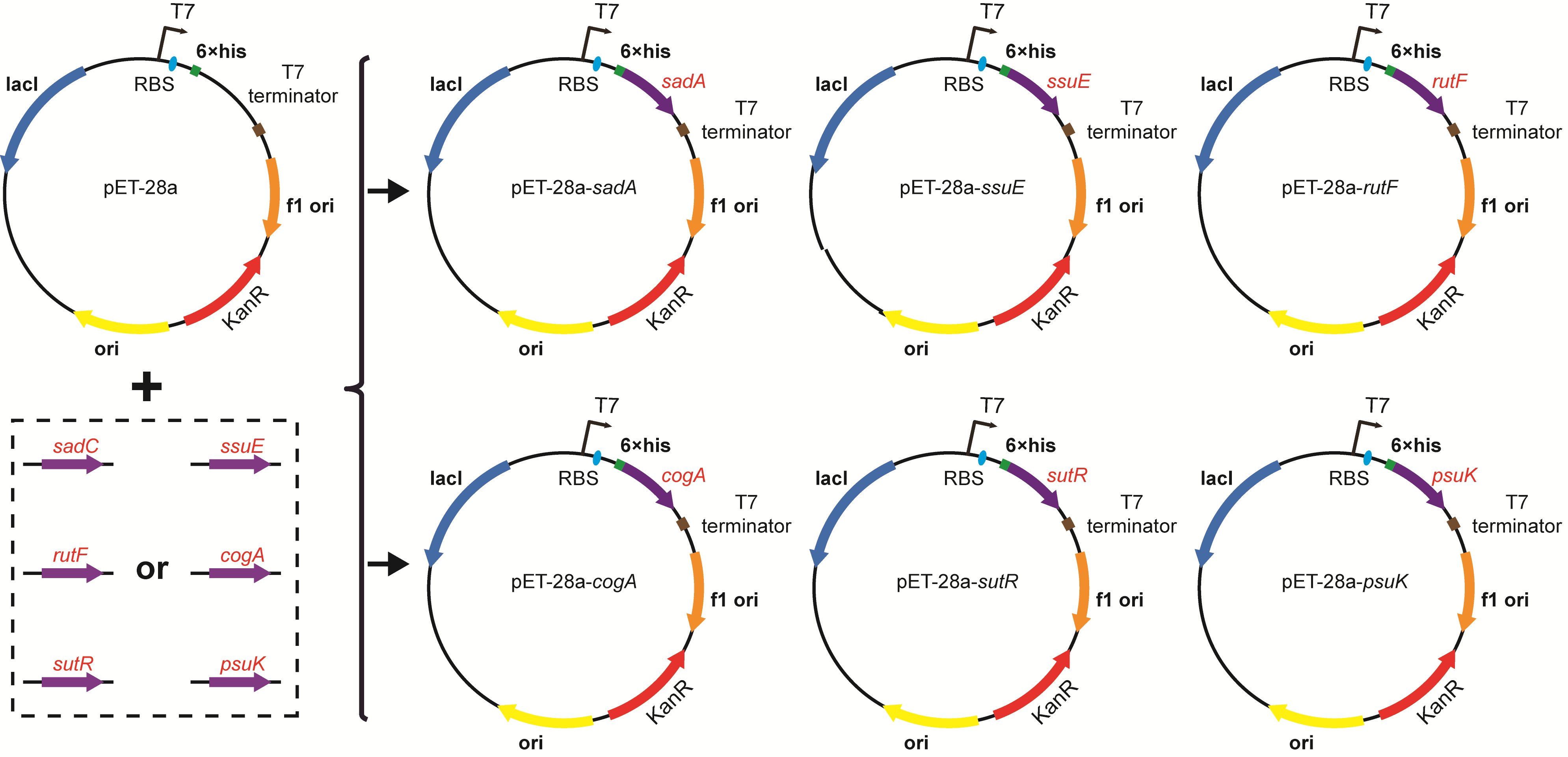


# Supplementary Figure 5. The plasmid schematic diagrams of plasmids with different FMN reductase genes.

The six plasmids carrying different FMN reductase genes were used to generate data in Figure 3D.


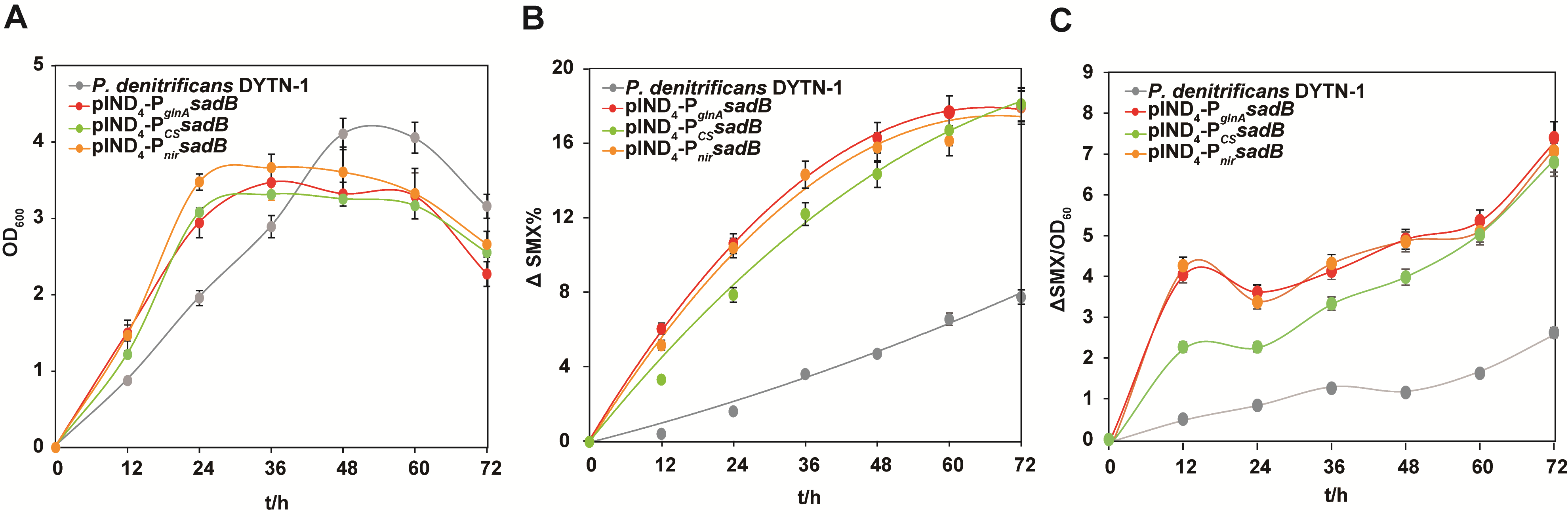


# Supplementary Figure 6. Time course of OD_600_ (A), ΔSMX (B), and ΔSMX/OD_600_ (C) with wild-type and *sadA* strains.


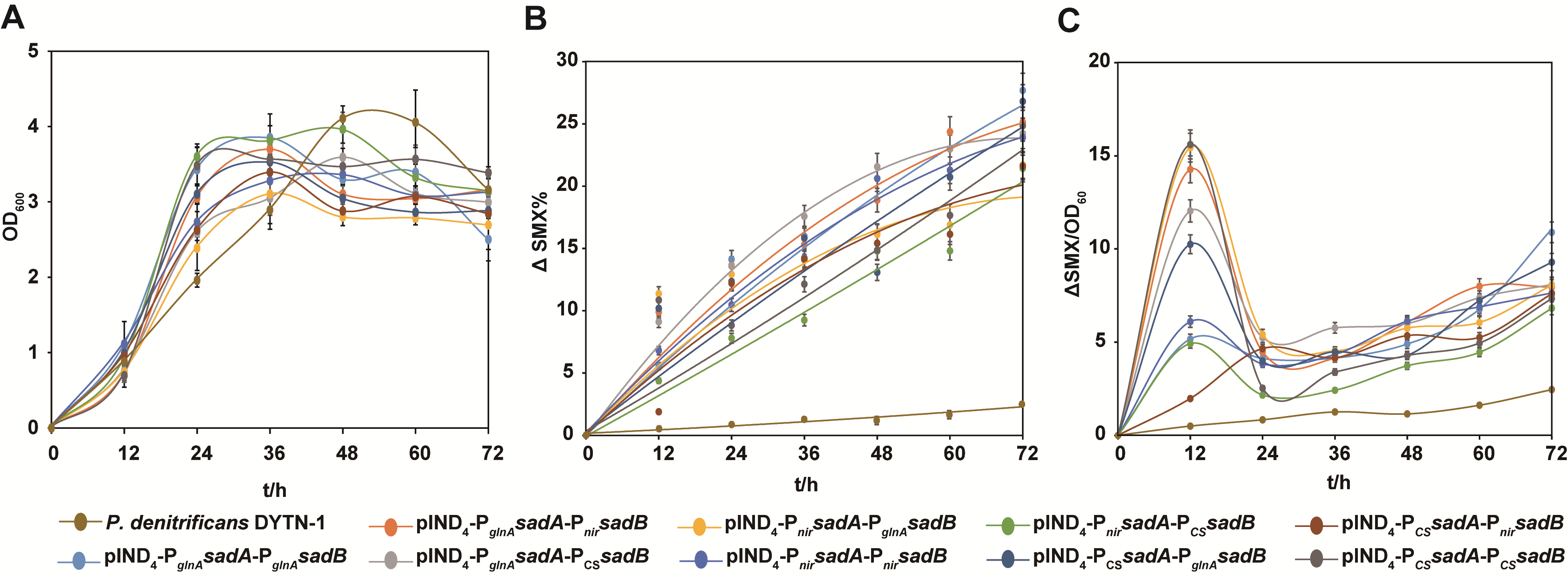


# Supplementary Figure 7. Time course of OD_600_ (A), ΔSMX (B), and ΔSMX/OD_600_ (C) with wild-type and *sadA*-*sadB* strains.


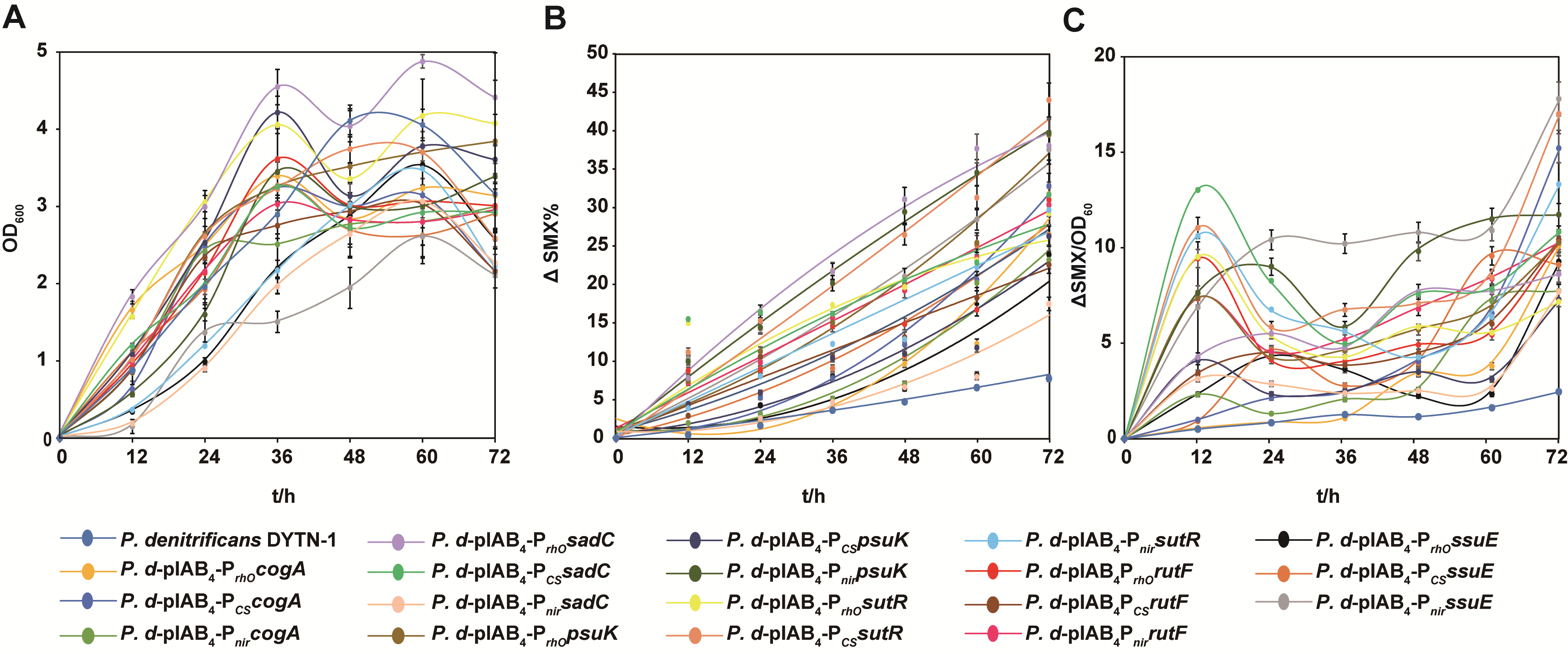


# Supplementary Figure 8. Time course of OD_600_ (A), ΔSMX (B), and ΔSMX/OD_600_ (C) with wild-type and *sadA*-*sadB-fmnR* strains.


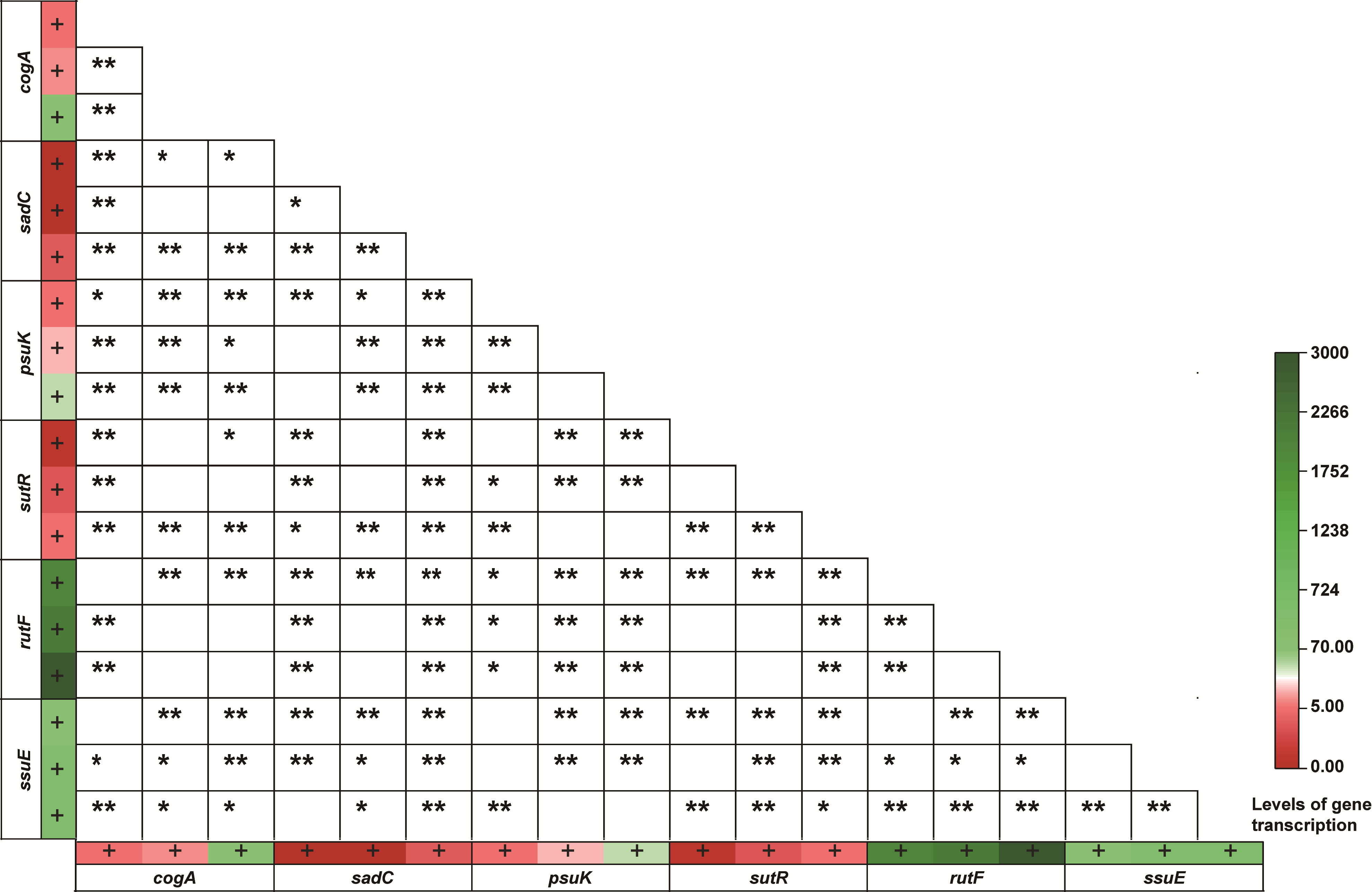


# Supplementary Figure 9. The Statistical analysis for degradation efficiency of *sadA*-*sadB-fmnR* strains.

“*” represents significant difference (0.01<*P*≤0.05), “**” represents highly significant difference (*P*≤0.01).


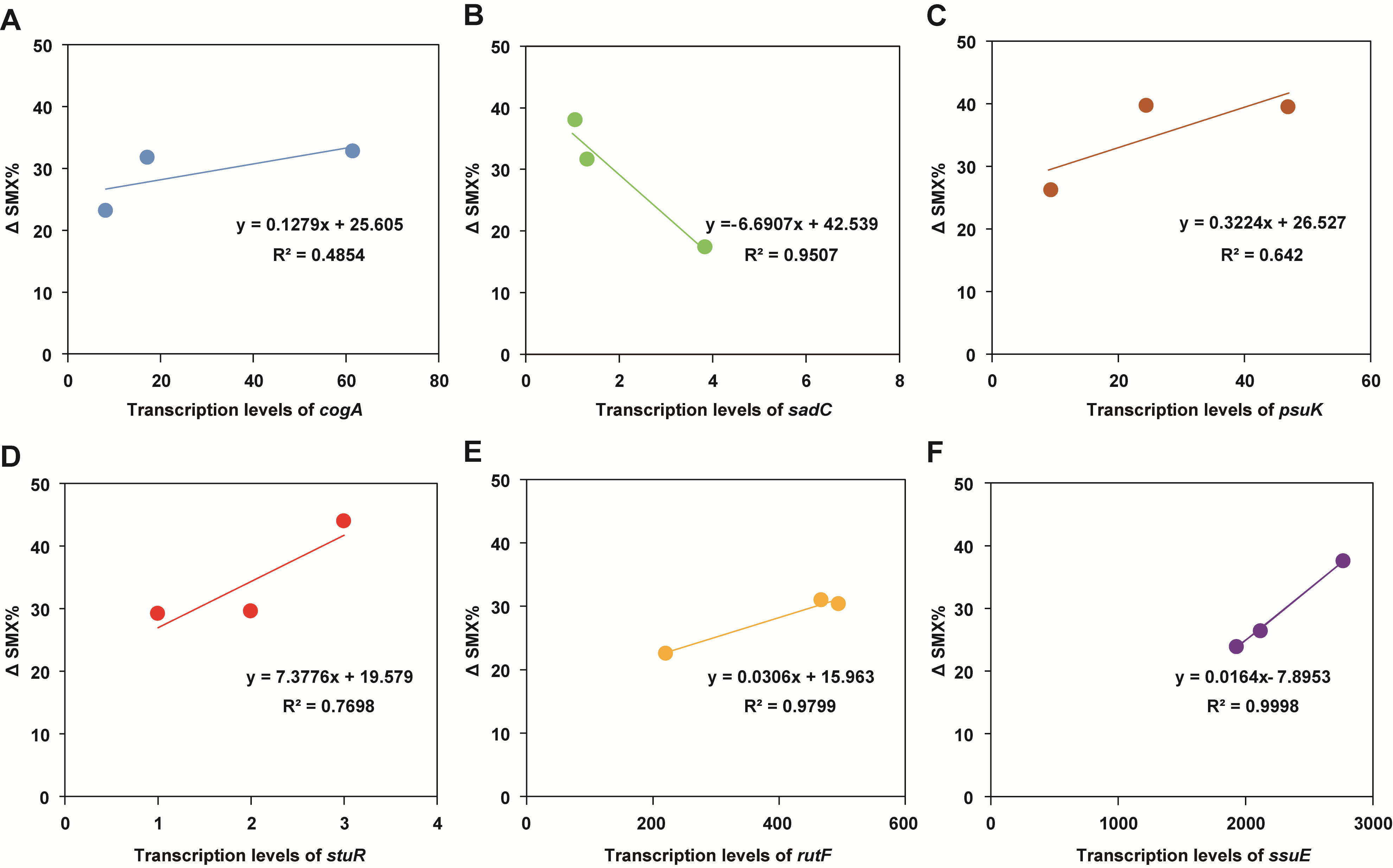


# Supplementary Figure 10. The scatter plot of degradation efficiencies with different levels of transcription in *cogA* (A), *sadC*(B), *psuK*(C), *stuR*(D), *rutF*(E), *ssuE*(F) strains.

# Supplementary Notes

The medium were prepared as following:

**Luria Bertani (LB) medium:** 10 g/L NaCl, 10 g/L tryptone, and 5 g/L yeast extract. For agar plates.

**M9 medium:** 4g/L Glycerin, 6.78g/L Na_2_HPO_4_, 3g/L KH_2_PO_4_, 0.5g/L NaCl, 1g/L NH_4_Cl, g/L MgSO_4_·7H_2_O g/L CaCl_2_.
